# Deep learning approaches for EEG-based healthcare applications: a comprehensive review

**DOI:** 10.3389/fnhum.2025.1689073

**Published:** 2026-01-23

**Authors:** RuiFang Lyu

**Affiliations:** School of Physical Education and Health, Geely University, Chengdu, China

**Keywords:** brain–computer interface (BCI), convolutional neural networks (CNNs), deep learning (DL), electroencephalography (EEG), healthcare, long short-term memory (LSTM), multimodal biosignal integration, recurrent neural networks (RNNs)

## Abstract

Electroencephalography (EEG) is a longstanding means of non-invasively recording brain signals and has become highly valuable for the study of neurological and cognitive processes. Recent progress in deep learning has also greatly improved both EEG signal analysis and interpretation, making more accurate, reliable and scalable solutions in various healthcare applications. In this review, we present a comprehensive summary of the convergence of EEG and deep learning, with an emphasis on diagnostic of neurological disorders, brain recovery, mental health conditions, and brain-computer interface (BCI) applications. We methodically investigate the application of convolutional neural networks (CNNs), recurrent neural networks (RNNs), long short-term memory (LSTM) models, transformer models and hybrid architectures for EEG-based tasks. Key challenges that have been hampering emerging solutions are critically covered, namely signal-related variability, the lack of data, and deep learning model limited interpretability. Finally, we highlight emerging trends, open issues and promising research directions, with the aim of laying a solid ground toward the improvement of EEG-based healthcare applications and to drive future research in this fast-growing research area.

## 1 Introduction

Among various non-invasive neuroimaging techniques, electroencephalography (EEG) is widely used due to its attributes such as high temporal resolution, portability, low cost and the ability of real-time measurements of brain activity. These characteristics make the EEG highly appropriate for clinical practice and research in various healthcare areas. In nervous system diseases, especially epilepsy, EEG is remains irreplaceable, and continues to be the standard reference in the detection of epileptiform discharges and in therapeutic decisions. The development of automated EEG interpretation and decoding systems has “rationalized” the diagnostic process, resulting in improved sensitivity as well as in reduced work load for the clinician, particularly in the context of critically ill patients admitted to intensive care units (ICUs) (Wu et al., 2024). Outside diagnostic neurology, EEG is vital in brain-computer interface (BCI) research and neural rehabilitation, since it can be used to develop assistive technologies such as EEG-controlled wheelchairs, communicative devices, and robotic rehabilitation systems for impaired individuals (Abiri et al., 2019). EEG-based BCI can be applied not only in controlled laboratory environments because of their non-invasive nature and easy deployment characteristics. In mental health and cognitive assessment, EEG has shown growing value in the identification and monitoring of disease such as depression, hyperactivity disorder, attention-deficit hyperactivity disorder (ADHD), schizophrenia and post-traumatic stress disorder (PTSD). EEG-derived biomarkers are associated with cognitive load, emotional state and attention and hold promise in early detection and longitudinal monitoring (Michel and Koenig, 2018). EEG also plays a key part in sleep and neurodevelopment research, due to its ability to describe oscillatory brain activity during sleeping cycle. EEG-based systems are currently used to diagnose sleep disorders, such as insomnia, narcolepsy and sleep apnoea (Stephansen et al., 2018; Roy et al., 2019).

For nearly a century, traditional analysis of EEG signals has been relying on classical mathematical methods and handcrafted feature extraction to read the brain activities. One of the most fundamental, well-established, and commonly used approaches is the Fast Fourier Transform (FFT). FFT allows to break EEG signals into its constituent frequencies, supporting therefore analysis over some standard frequency bands such as delta (0.5–4 Hz), theta (4–8 Hz), alpha (8–13 Hz), beta (13–30 Hz) and gamma (>30 Hz) (Atangana et al., 2020). Besides the FFT, wavelet transform is extensively employed in time-frequency analysis, and produces a better localization of transient and non-stationary features in EEG. The wavelet transform allows multi-resolution analysis by dividing signals into components spatially localized in both time and frequency, rendering it particularly suitable for modelling short, event-related transients such as epileptic spikes. Another classical approach of extraction is auto-regressive (AR) modelling which employs time series analysis in order to estimate the spectral content of EEG for feature extraction. AR may offer better frequency resolution than FFT, but it depends on the signal being stationary and on the model order selection (Nor et al., 2022). Statistical descriptors, such as power spectral density (PSD), entropy measures, Hjorth parameters, and coherence analysis forms the mainstay of traditional EEG processing pipelines for feature extraction. These features are subsequently given as input to classical machine learning classifiers (e.g., support vector machines (SVMs), linear discriminant analysis (LDA), and k-nearest neighbors (k-NN), etc.) for seizures detection, sleep staging, motor imagery classifications and etc. (Lotte et al., 2018). Although traditional approaches have made great progress, they are highly relied on hand-crafted feature extraction and domain experience, which are labour-intensive and may have subjective bias.

Moreover, while FFT and AR methods assume the stationarity of signals, EEG signals deviate from that assumption because of the high variability and non-linearly non-stationary their nature (Acharya et al., 2018). Therefore, these techniques may not be able to capture the complex spatiotemporal dynamics of the underlying brain activity. Despite these problems, factors that are observed across all applications and algorithms include the problem of low quality or low signal-to-noise ratio (SNR), non-stationarity, and massive inter-subject variability. These reasons have made the conventional signal-processing approach and shallow machine learning difficult to produce good results in the clinical setting.

**Figure 1:**
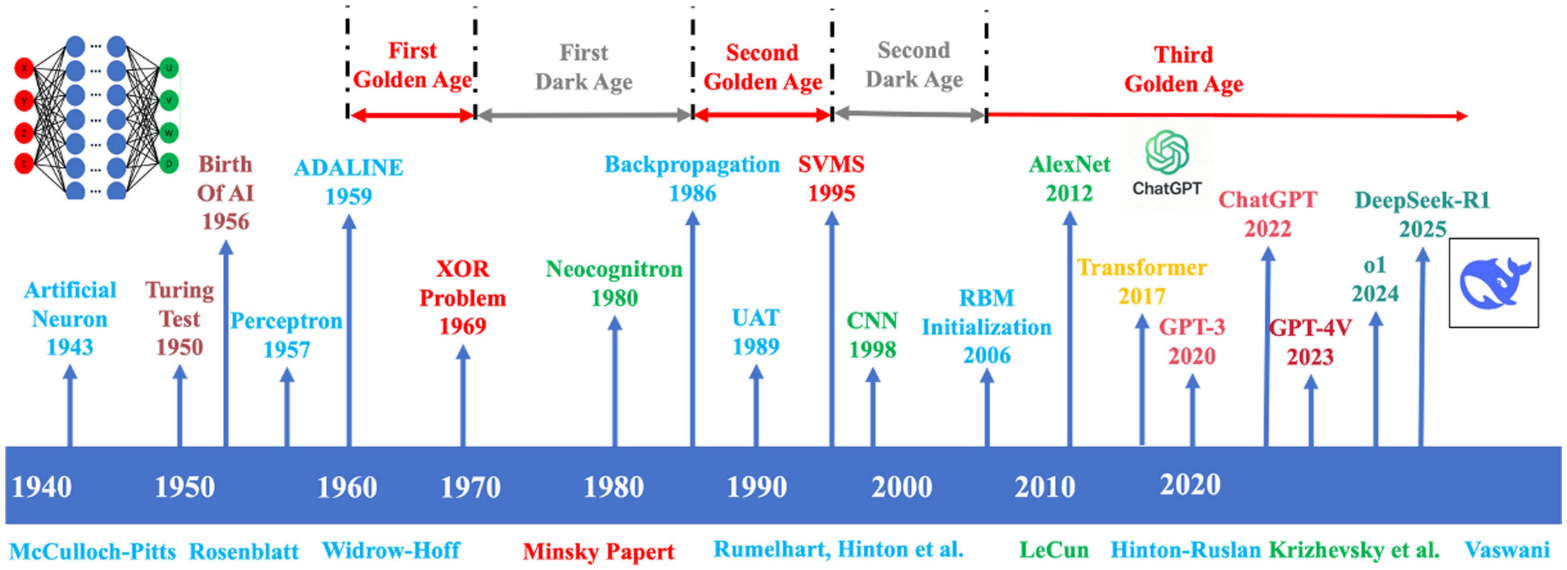
Evolution timeline of deep learning architectures.

Recent developments in deep learning (DL) have led to remarkable progress for both EEG signal classification, feature extraction and real-time decoding (Roy et al., 2019). In the past years, the EEG analysis has evolved from manual, conventional signal processing to DL methods (Figure 1). EEG based health-care applications in recent years have been centralising around DL essentially because the network is able to directly learn and recognize more intricate patterns from raw and from lightly pre-processed signals. The possibility of knowledge and technology transfer has contributed to the significant improvement in the diagnostic performances, device reliability, and its applicability within different clinical environments. Convolutional neural networks (CNNs) are one of the most popular deep-learning architectures, as they are known to effectively capture spatial information and learn hierarchies of feature representations. The latter property makes CNNs particularly useful for abnormal EEG activity detection, such as epileptic seizures, in which distributed anomalies need to be detected with high accuracy (Roy et al., 2019). CNNs have also shown excellent performance in automatic sleep stage scoring, even comparable to those of experienced human scorers (Kobayashi et al., 2023). Temporal information is inherent to EEG signals, however, and models that can preserve sequential dependencies are also important. Recurrent neural networks (RNNs), in particular, long short-term memory (LSTM) networks, have made remarkable progress in solving it. Unlike standard analysis approaches to which LSTM are compared, fixed-length windows are not used to present the data to the network and instead LSTM use EEG as a continuous temporal sequence which captures brain dynamics more effectively (Wu et al., 2020). Furthermore, CNNs and hybrid CNNs-based models have reported significant achievement in discriminating subtle ictal and interictal pattern differences, often exceeding those of classical techniques and offering clinicians useful tools for early seizure intervention (Roy et al., 2019). The neurodegenerative diseases have also made their progress. For Alzheimer’s disease (AD) and mild cognitive impairment (MCI), deep neural networks applied to EEG rhythms and connectivity measures have enabled the identification of early pathological changes that are often overlooked in standard clinical evaluations (Morovati et al., 2024). Early detection of such changes is important to facilitate early therapeutic interventions. In addition to neurological diseases, DL has also displayed impressive prospects in sleep medicine. Another significant application is automated sleep staging and the diagnosis of sleep disorders. Sleep stages are classified using CNNs- and LSTM-networks and the final prediction results are comparable to those of human experts whilst allowing for cost-effective, large-scale screening for diseases such as insomnia, sleep apnoea or narcolepsy (Almutairi, 2024). Through utilizing temporal and spectral EEG features these models optimize computational time, standardize analysis and interpretation for use in clinical diagnostic reinforcement. EEG-DL fusion has also played its role in mental health diagnosis. They have been used to objectively confirm illness-related patterns of activity in disorders like depression, anxiety, and schizophrenia, which were otherwise assessed using subjective interviews. Using both recurrent and transformer-based models, studies have been able to identify dynamic modulations in brain activity during emotional and cognitive states, opening up the possibility for novel biomarkers of diagnosis and treatment monitoring (Wan et al., 2023).

DL has also accelerated BCI development, particularly for severe motor impairments. CNN-LSTM models integrated with attention mechanisms now enable real-time decoding of motor imagery and cognitive commands, driving assistive technologies like robotic limbs, wheelchair controls, and communication devices. It is directly enhanced user independence and quality of life (Tayeb et al., 2019; Huang et al., 2018). Beyond clinical domains, EEG-driven DL demonstrates value in occupational health, where monitoring cognitive load and fatigue through EEG analysis enables real-time alert systems for accident prevention in high-risk sectors such as transportation and industrial operations (Morton et al., 2022). Further expanding applications, multimodal frameworks integrating EEG with bio-signals and clinical metadata show promise for improving diagnostic precision and outcome prediction in complex conditions such as stroke (Choi et al., 2021). By synthesizing complementary physiological data, these approaches advance personalized, holistic health assessments. The progression of DL continues to transform EEG-based healthcare—delivering automated, accurate, and scalable analysis—with growing integration into clinical workflows and wearable diagnostics.

**Figure 2:**
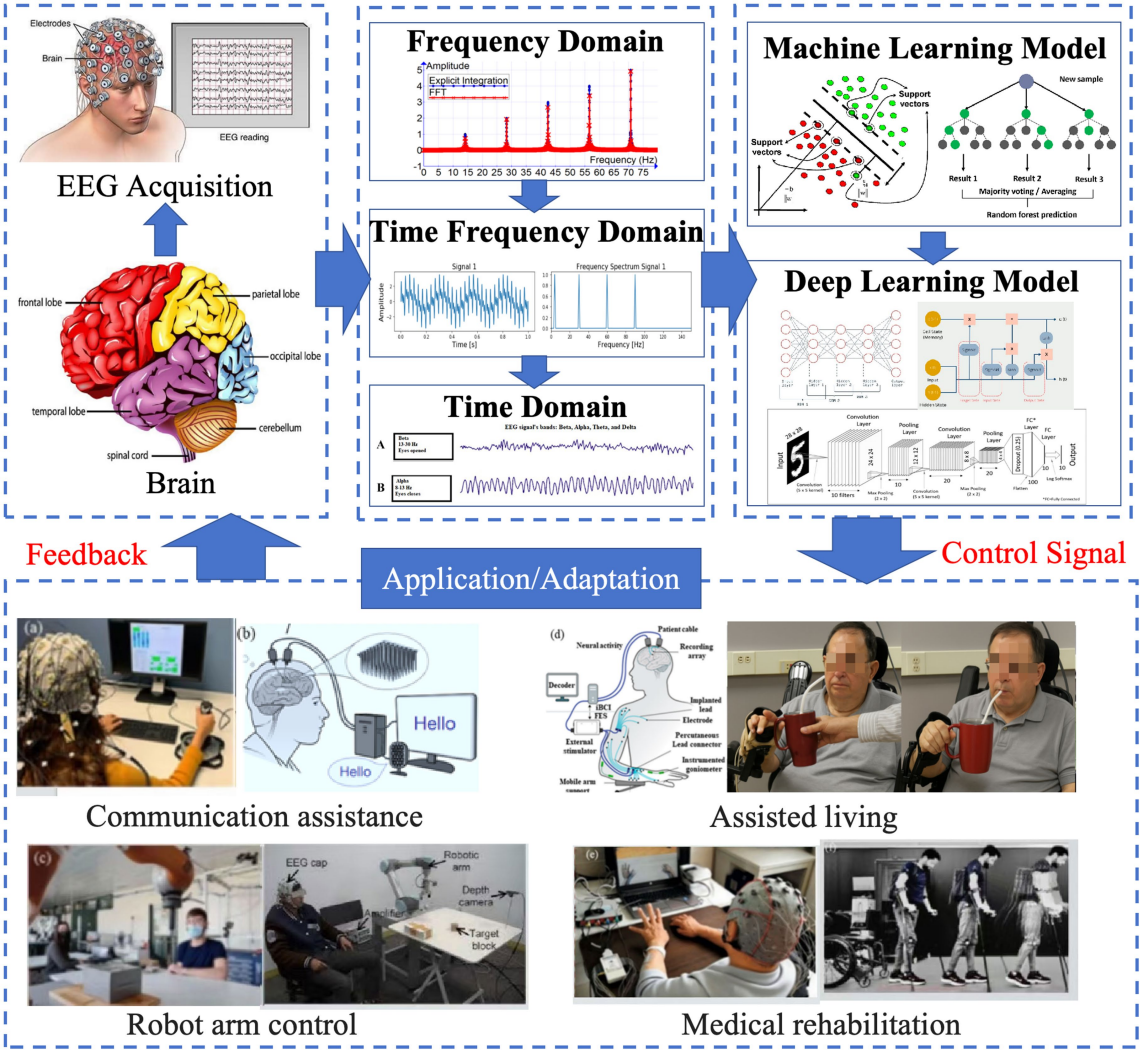
Typical workflow of DL–driven EEG healthcare applications.

This review presents a comprehensive synthesis of DL approaches applied to EEG-based healthcare, highlighting recent advances, current limitations, and future research directions. It encompasses core aspects of EEG signal processing, state-of-the-art DL architectures, and their applications in the diagnosis and monitoring of neurological, psychiatric, and cognitive disorders. Emerging trends are discussed as key enablers for next-generation healthcare technologies, such as transfer learning, multimodal data integration, and real-time deployment in wearable or clinical systems. Figure 2 presents the general workflow of DL–driven EEG healthcare applications, which serves as the conceptual backbone for this review. The pipeline begins with EEG acquisition and preprocessing, proceeds through feature extraction and DL model development, and culminates in clinical or healthcare deployment. The review is structured to benefit both newcomers and experienced researchers. Section 2: details the PRISMA-based search strategy, inclusion/exclusion criteria, and stage definitions that structure the remainder of the review. Section 3: Background and Fundamentals outlines EEG characteristics, conventional signal processing techniques, and the primary analytical challenges. Section 4: DL Architectures for EEG analyzes major model types—including CNNs, RNNs, LSTM networks, transformers, and hybrid designs—with emphasis on strategies tailored to EEG’s spatial–temporal dynamics. Section 5: Healthcare Applications surveys deployments in epilepsy detection, sleep disorder classification, mental health assessment, BCI systems, cognitive workload monitoring, and neurorehabilitation. Section 6: Challenges and Future Directions addresses key barriers—such as data scarcity, limited interpretability, inter-subject variability, low signal-to-noise ratio, and computational constraints—while highlighting promising avenues for advancing robustness and clinical translation. Section 7: Conclusion synthesizes key insights and reaffirms the transformative role of DL in EEG-based healthcare. By integrating these perspectives, the review aims to guide future research and promote the adoption of DL–enhanced EEG systems in real-world medical practice.

This article is a conceptual and methodological review of DL approaches for EEG-based healthcare. We deliberately do not cover implementation- or protocol-level details such as input tensor formats and dimensions, dataset splitting criteria, leakage prevention rules, cross-validation schemes, early stopping, or class-imbalance management. Our aim is to synthesize architectural trends, representation learning paradigms, and evaluation principles at a high level. Readers seeking practical guidance for end-to-end pipelines are referred to widely used open-source ecosystems (e.g., MNE-Python/Braindecode) and benchmarking frameworks (e.g., Mother of All BCI Benchmarks, MOABB), which provide concrete examples and reproducible templates.

## 2 Methodology

This review was conducted using the Preferred Reporting Items for Systemic Reviews and Meta-Analyses (PRISMA 2020 guideline) (Page et al., 2021). Comprehensive searches were run in Web of Science, PubMed, IEEE and Science Direct from 2015 to 2025. Reference lists of included studies and recent topic reviews were hand-searched. Queries combined four concept blocks with Boolean operators and field tags adapted per database: (EEG) AND (deep learning/architecture keywords: CNNs, RNNs/LSTM, Transformer/attention, GNNs) AND (application keywords: clinical/healthcare tasks such as seizure, sleep staging, mental, BCI, rehabilitation). In addition, the list of citations and references of each paper was looked over to check for any relevant studies that could be used. We included peer-reviewed journal articles or full-length conference papers in English that used EEG and deep learning for healthcare-relevant tasks (e.g., classification, detection, prediction, reconstruction, or representation learning). We excluded studies that did not employ deep learning; were not in English; were publication types other than full-length, peer-reviewed journal articles or conference papers (e.g., conference summaries, books/book chapters, or other non-journal formats); lacked quantitative evaluation metrics; were reviews, systematic mappings, or survey papers; addressed applications outside our target scope; or otherwise failed to meet the prespecified inclusion criteria.

**Figure 3:**
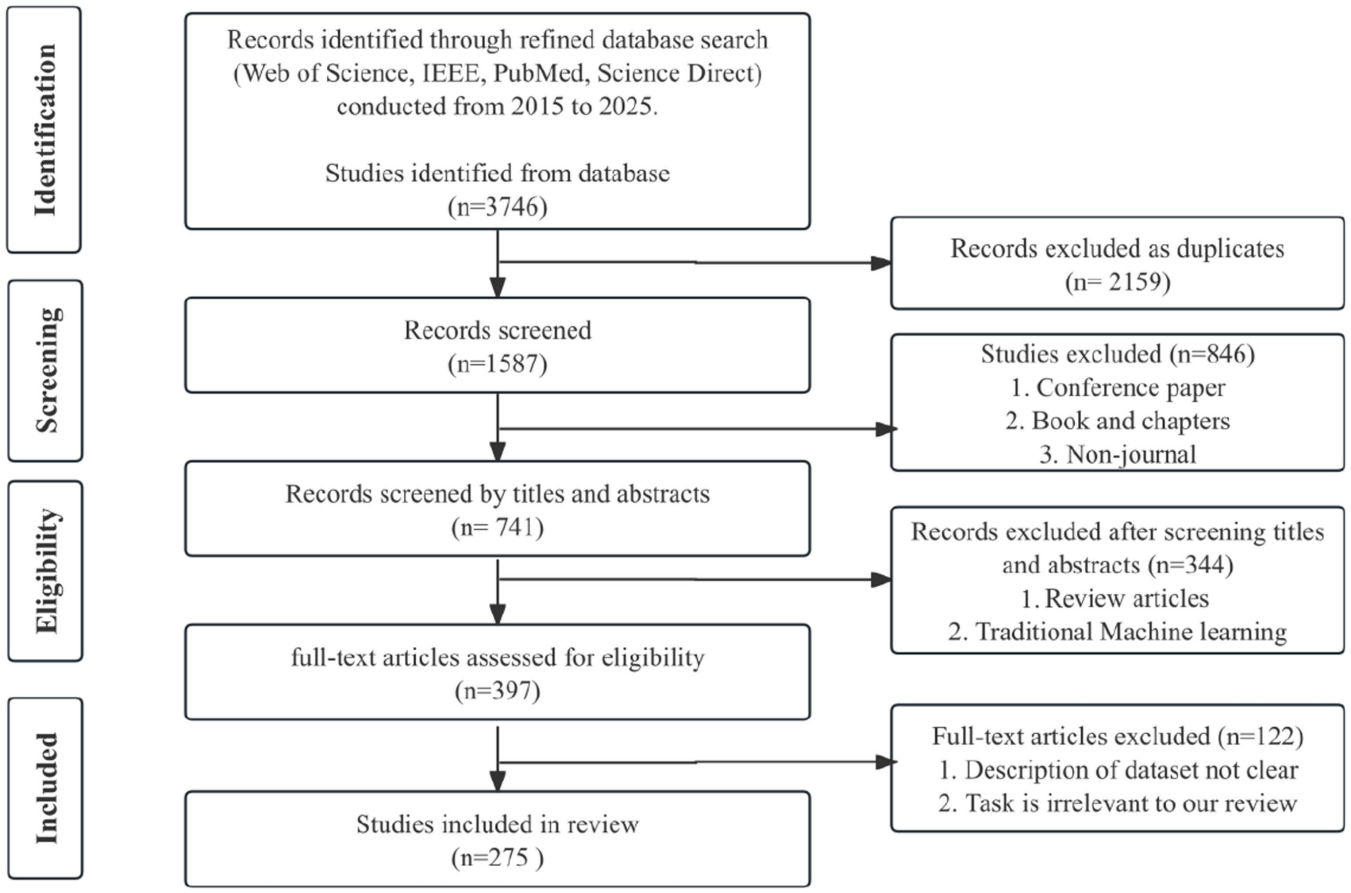
PRISMA study selection diagram with four stages of the PRISMA study selection process: identification, screening, eligibility, and inclusion.

Figure 3 summarizes the study selection. Following PRISMA, the initial search across all databases yielded 3,746 records. After removing 2,159 duplicates, 1,587 unique records remained. Before title/abstract screening, we excluded 846 items due to ineligible publication types (conference summaries/short papers, books or book chapters, and other non-journal formats), leaving 741 records for screening. Two reviewers independently screened all titles and abstracts, with a third resolving disagreements. This step excluded 344 records (reviews and traditional machine-learning papers), and 397 articles were retrieved for full-text assessment. At the full-text stage, 122 records were excluded, resulting in 275 studies included in the review.

## 3 Background

### 3.1 Overview of EEG signal acquisition and characteristics

EEG is a widely used noninvasive neuroimaging technique that records the brain’s electrical activity via electrodes placed on the scalp surface. This activity originates from the synchronous firing of large populations of cortical neurons, producing voltage fluctuations that EEG captures with high temporal resolution, typically in the order of milliseconds (Franco i Moral, 2025). Due to its noninvasive nature, portability, and relatively low cost compared to other neuroimaging modalities such as functional magnetic resonance imaging (fMRI) or magnetoencephalography (MEG), EEG remains a cornerstone technology for clinical diagnosis, cognitive neuroscience research, and brain-computer interface development.

EEG signal acquisition begins with the placement of electrodes according to standardized montages, such as the International 10–20 or 10–10 systems. These configurations define fixed scalp positions relative to anatomical landmarks, ensuring reproducibility and consistent spatial coverage across cortical regions (Moutonnet et al., 2024). Electrodes may be either wet—requiring conductive gel to reduce impedance and improve signal quality—or dry, which offer greater ease of use and patient comfort but may introduce higher impedance and susceptibility to noise. The choice of electrode type is often dictated by the specific application, requiring a trade-off between signal fidelity and practicality. Furthermore, the electrical potentials recorded by EEG are inherently weak, typically ranging from 1 to 100 microvolts, necessitating the use of low-noise, high-gain amplifiers to enhance signal strength while preserving signal integrity (Chen et al., 2022). Contemporary EEG systems often incorporate active electrodes and wireless communication technologies to improve signal quality, reduce motion artifacts, and enhance usability in mobile or clinical environments.

The EEG frequency bands reflect the dynamic functional states of underlying neuronal populations and are modulated by both physiological processes and pathological conditions. A key characteristic of EEG signals is their nonstationary and nonlinear nature, which reflects the brain’s complex and continuously evolving activity in response to both internal cognitive processes and external environmental influences (Houssein et al., 2022). This inherent variability creates considerable challenges for conventional signal processing techniques, many of which depend on the assumption of stationarity. Consequently, more advanced computational strategies, such as adaptive filtering, are often required to effectively capture and interpret EEG dynamics. With respect to spatial characteristics, EEG provides important insights into the distribution of neural activity across the scalp, though its spatial resolution remains limited compared with invasive or high-resolution neuroimaging techniques. To address this limitation, EEG is often combined with structural imaging modalities such as magnetic resonance imaging (MRI) to facilitate source localization. This multimodal integration improves the clinical interpretability of EEG data, particularly in epilepsy, where precise identification of epileptogenic zones is critical for accurate diagnosis and effective treatment planning (Sahlas et al., 2024).

### 3.2 Preprocessing techniques for EEG

EEG signals are highly susceptible to noise and artifacts, which can markedly reduce the reliability and accuracy of subsequent analysis and classification. Preprocessing is therefore a crucial step to improve signal quality and ensure that extracted features are dependable for downstream analytical tasks. A series of preprocessing procedures are typically applied to mitigate noise and artifacts while also standardizing and segmenting the data for efficient feature extraction. These procedures are essential for enhancing the SNR, enabling comparability across trials and subjects, and preparing the data for DL models. Table 1 summarizes the primary preprocessing techniques commonly employed in EEG studies, including filtering, artifact removal, baseline correction, segmentation, normalization DL–based preprocessing. The integration of these methods renders EEG signals more suitable for feature extraction and classification, thereby strengthening the robustness and accuracy of deep learning approaches in practical applications.

**Table 1:** Preprocessing techniques for EEG.

Step	Purpose	Typical methods	Notes
Filtering	Remove noise and isolate relevant frequency bands	Bandpass filters (0.5–40 Hz), notch filters (50 Hz)	Removes slow drifts, muscle noise, and electrical interference
Artifact Removal	Remove physiological and environmental artifacts	Independent Component Analysis (ICA), wavelet-based techniques, thresholding	Critical for accurate analysis; handles EOG, EMG, ECG artifacts
Baseline Correction	Mitigate slow drifts and stabilize signal baseline	Subtract mean/median value from pre-stimulus interval	Ensures consistency across trials
Segmentation (Epoching)	Divide continuous EEG into time-windows for analysis	Event-related potential (ERP) analysis	Facilitates time-specific feature extraction
Normalization/Scaling	Standardize data for better model convergence	Z-score normalization, min–max scaling (Kobayashi et al., 2023)	Reduces inter-subject variability and improves classification
Automated Pipelines	Integrate preprocessing steps for efficiency	EEGLAB, MNE-Python	Standardizes workflows, improving reproducibility
DL-based preprocessing	Data-driven artifact detection, denoising, and reconstruction	CNN artifact classifiers; dual-scale CNN-LSTM reconstructors; GAN/Transformer denoisers	Preserves ERP timing and oscillatory structure; robust to non-stationary artifacts; requires representative training data

An essential step in EEG preprocessing is filtering, which isolates relevant frequency bands while attenuating unwanted noise. Bandpass filters, typically ranging from 0.5 Hz to 40 Hz, are widely used to remove slow baseline drifts and high-frequency disturbances such as muscle artifacts and environmental electrical interference (Kalita et al., 2024). Notch filters, commonly set at 50 Hz depending on local power line frequency, are further applied to suppress line noise (Alkahtani et al., 2023). Artifact detection and removal represent another critical component, targeting physiological sources including eye blinks and movements (electrooculogram, EOG), muscle activity (electromyogram, EMG), and cardiac signals (electrocardiogram, ECG). Since these artifacts often overlap with EEG frequency ranges, failure to address them can obscure neural information. Mitigation strategies vary from simple threshold-based rejection to advanced blind source separation techniques, most notably Independent Component Analysis (ICA), which decomposes EEG into statistically independent components to identify and eliminate artifact-related sources (Mannan et al., 2018). Wavelet-based approaches have also been employed to selectively suppress artifacts while retaining the underlying neural signals. Baseline correction is commonly applied to counteract slow drifts by subtracting the mean or median of a pre-stimulus interval, thereby stabilizing the baseline and enabling comparisons across trials and participants. Finally, Segmentation, or epoching, divides continuous EEG recordings into shorter, event-locked intervals, allowing event-related potential (ERP) analysis and time-specific feature extraction. Appropriate epoch selection is crucial to balance assumptions of signal stationarity with the inclusion of relevant brain responses.

Normalization and scaling are routinely implemented to standardize feature ranges and support model convergence in machine learning workflows. Common methods, such as z-score normalization and min-max scaling, help reduce inter-subject variability and enhance classification performance (Degno et al., 2021; Flanagan and Saikia, 2023). More recently, automated preprocessing pipelines have been developed to integrate multiple steps into streamlined workflows, improving reproducibility and efficiency. Widely used open-source toolboxes, such as EEGLAB and MNE-Python, provide comprehensive capabilities for filtering, artifact correction, segmentation, and feature extraction, and have become established resources in EEG research (Das et al., 2023).

DL methods have also been developed to enable supervised artifact identification, context-aware reconstruction, and based on Generative Adversarial Networks (GAN) denoising that enhance signal fidelity and task performance. Supervised artifact identification uses CNNs to classify or localize contamination from raw segments or time–frequency maps; in intracranial EEG, this enables high-fidelity flagging and masking of non-neural activity before analysis (Nejedly et al., 2019). Reconstruction models replace manual component selection by learning to recover clean signals: dual-scale CNN-LSTM hybrids preserve ERP morphology and oscillatory structure while suppressing EMG/EOG (Gao et al., 2023). Under heavy noise or distribution shift, adversarial approaches (e.g., Dual-Branch Hybrid CNN-Transformer-based GAN) couple CNNs with Transformers to fuse local and global context; adversarial training sharpens reconstructions and preserves spectral landmarks without filter-induced ringing (Cai et al., 2025). Recommended practice is a hybrid pipeline: minimal classical front-end (detrend, band-pass, notch, re-reference), CNN-based artifact scoring, targeted denoising via CNN-LSTM or GAN/Transformer, then spectral/phase validation before normalization and segmentation.

### 3.3 Challenges in EEG analysis (non-stationarity, low SNR, inter-subject variability)

EEG signals are extensively used in healthcare applications due to their noninvasive nature and high temporal resolution. Nevertheless, several intrinsic properties of EEG data pose substantial challenges to accurate analysis and interpretation. One of the most prominent issues is signal non-stationarity, as the statistical characteristics of EEG fluctuate over time in response to dynamic neural activity, changes in cognitive state, fatigue, and external perturbations (Núñez et al., 2020). Such temporal variability can markedly constrain the generalizability of machine learning models that operate under the assumption of stationary input within fixed time segments (Banville, 2022). Another critical limitation is the intrinsically low SNR. EEG amplitudes typically fall within the range of 10–100 μV and are highly vulnerable to interference from both physiological sources, such as eye blinks, muscle activity and environmental noise, including power-line artifacts (Mannan et al., 2018). This challenge is particularly acute in real-time healthcare monitoring, where sustaining a high SNR remains a major obstacle (Roy et al., 2019).

Inter-subject variability represents a significant challenge in EEG analysis, as neural activity patterns can vary widely among individuals due to differences in neuroanatomy, cognitive function, and mental state. Such variability complicates the development of generalized diagnostic models, since algorithms trained on one subject’s data often perform less accurately when applied to others. Techniques such as transfer learning and domain adaptation have shown potential in addressing this issue; however, these strategies remain an active area of investigation and are still being refined (Zhang et al., 2021). Overcoming these challenges will require the development of robust algorithms that can adapt to non-stationary signals, enhance signal quality, and maintain stable performance across heterogeneous populations.

## 4 Deep learning architectures for EEG-based signal recognition

DL-based approaches have been widely developed for EEG-based signal recognition and can be classified into different categories, including CNN- and Graph Neural Network (GNN)-based approaches to extract features from spatial data, RNN/LSTM methods for handling the temporal dependencies of EEG signals, Transformer-based methods with attention mechanisms, and hybrid architectures. In addition, we also discuss the role of transfer learning and adversarial learning to further improve the effectiveness of these DL-based approaches for EEG-based signal recognition. Across EEG recognition, CNNs excel at learning local spatial-spectral motifs from raw maps/topomaps with small compute and strong edge deployability, but they underperform when long temporal context or cross-channel dependencies dominate and can overfit limited cohorts; RNNs/LSTMs capture sequence dynamics and sleep/ictal transitions yet train slowly, suffer vanishing gradients on long windows, and are fragile to class imbalance; Transformers, with self-attention, model global, cross-channel relations and increasingly set state-of-the-art in affect/workload and multi-event decoding, but demand larger data, careful regularization, and higher latency; hybrids (CNN-LSTM, CNN-Transformer/Conformer) pragmatically fuse local and global cues, improving robustness at the cost of complexity and tuning burden; GNNs encode electrodes as graphs to exploit connectivity and yield interpretable attributions, though performance hinges on graph construction and risks overfitting with small, heterogeneous datasets; transfer learning reduces subject/session calibration but can suffer negative transfer and requires shift-detection/reporting; finally, self-supervised/contrastive or masked pre-training on unlabeled multi-center EEG consistently improves few-label generalization and “thinker-invariance” provided augmentations are physiologically plausible and leakage-safe.

**Figure 4:**
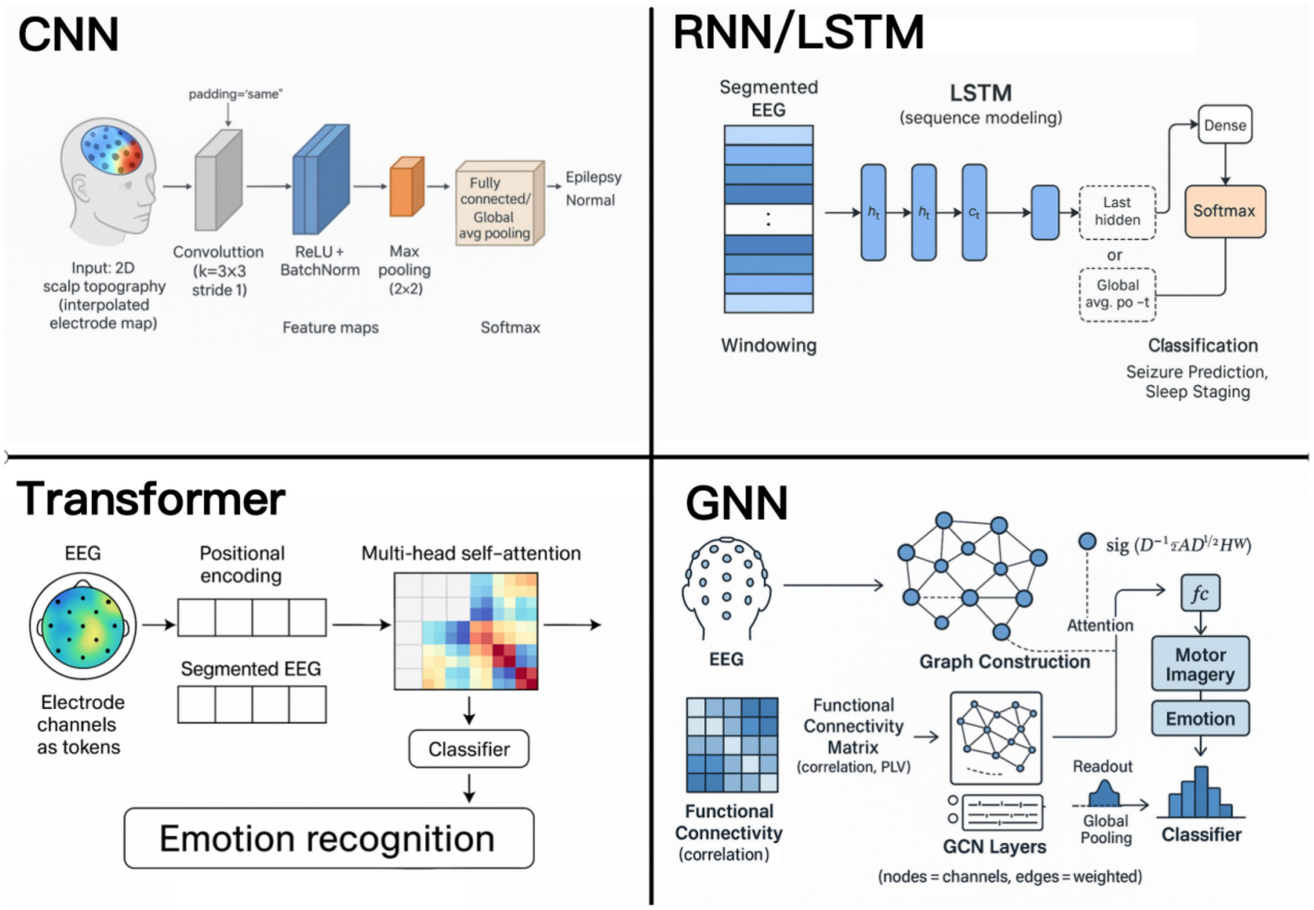
Deep learning architectures for EEG.

Figure 4 provides a schematic overview of four fundamental network architectures: CNNs, RNNs/LSTM, Transformers, and GNNs. CNNs-based approaches exploit spatial dependencies among electrodes depicting temporal information, resulting in superior performance in applications like epilepsy detection, where these dependencies are significant in distinguishing normal and abnormal patterns. RNNs and LSTM models are especially well-suited to model temporal dependencies in EEG sequences, allowing the capture of dynamic sequences such as those encountered in sleep stage classification. Transformer models utilize self-attention to model long-range dependencies between electrode channels, and have shown great success in, e.g., emotion recognition. GNNs regard EEG electrodes as the nodes in a connectivity graph that is able to describe the relationships between channels, such as for motor imagery decoding or BCI control. This variety in architectural choices is a demonstration of the flexibility of deep-learning frameworks for diverse EEG research goals and application areas. Table 2 summarizes the most popular DL architectures used in healthcare applications and its features in EEG domain. This table offers a brief comparison on aspects of key characteristics, complex, input latitude, application fields, strengths, weaknesses, and representative studies, which acts as a handy benchmark for choosing relevant models (Sasatake and Matsushita, 2025; Weng et al., 2025).

**Table 2:** Summary of deep learning architectures used in EEG-based healthcare.

Architecture	Key features	Complex	Input latitude	Typical applications	Strengths	Limitations	Representative studies
CNN	Spatial feature extraction, convolution layers	Low–Med	High rep., Med length/channels, Low topology.	Seizure detection, sleep staging, BCI	Captures local spatial patterns, less manual feature engineering	Needs large datasets, weak temporal modeling	Acharya et al. (2018) and Supratak et al. (2017)
RNN/ LSTM	Sequential modeling, temporal dependencies	Med–High	Med rep., High length, Low–Med channels, Low topology.	Emotion recognition, seizure prediction	Models long-term dependencies	Sensitive to data imbalance, slower training	Tripathi et al. (2017)
Transformer	Self-attention, parallel processing	High	High rep./length, Med channels, Low topology	Cognitive workload estimation, emotion recognition	Captures global dependencies, interpretable attention	Computationally heavy, needs large data	Klein et al. (2025)
CNN–LSTM Hybrid	Combines spatial + temporal modeling	Med–High	High rep./length, Med channels, Low topology	Seizure detection, sleep staging	Joint spatio-temporal learning	High computational cost	Roy et al. (2019)
GNN	Graph-based electrode modeling	Med–High	Med rep./length, High channels/topology	Motor imagery, emotion recognition	Captures inter-channel connectivity	Graph construction complexity	Xu et al. (2024)

### 4.1 CNNs for spatial feature extraction

CNNs is a feed-forward neural architecture widely used in image processing and natural language processing (NLP), and it has also demonstrated strong applicability in time series prediction tasks (Rajakumari and Lanjewar, 2025). Benefiting from its local receptive fields and weight sharing mechanism, CNNs significantly reduces the number of trainable parameters, thereby enhancing learning efficiency compared to fully connected architectures. To formally describe the convolution process, let the input tensor be $X\in {\mathbb{R}}^{H\times W\times C}$ , where H, W and C denote the height, width, and number of channels, respectively. The convolution operation in Equation (1) is defined as:


$$Y(i,j)=\sum_{m=1}^{k_{h}}\sum_{n=1}^{k_{w}}\sum_{c=1}^{C}X(i+m-1,j+n-1,c)\cdot K(m,n,c)+b$$ (1)

where $K\in {\mathbb{R}}^{k_{h}\times k_{w}\times C}$ represents the convolution kernel and *b* is the learnable bias term. The indices *i* and *j* denote spatial coordinates of the output feature map. When stride s and padding p are adopted, the output tensor size in Equation (2) is computed as:


$$\;H\prime =\frac{H-k_{h}+2p}{s}+1,W\prime =\frac{W-k_{w}+2p}{s}$$ (2)

Following each convolution, a nonlinear activation function is applied to enhance the network’s representation capacity. A commonly used choice is the rectified linear unit (ReLU), in Equation (3) is defined as:


ReLU(x)=max(0,x) (3)

To enhance robustness to small spatial variations and reduce computational cost, max-pooling layers are incorporated between convolutional blocks. The network concludes with one or more fully connected layers in Equation (4) is defined as:


y=Wx+b (4)

where *W* and *b* denote the weight matrix and bias vector, respectively. This hierarchical architecture enables the CNNs to capture low-level textures in early layers and high-level semantic patterns in deeper layers, resulting in compact feature embeddings.

CNNs have become increasingly prominent in EEG signal analysis due to their ability to automatically learn spatial feature hierarchies from raw or preprocessed data. Although first developed for image recognition, CNNs are particularly adept at capturing local spatial dependencies, making them well-suited for interpreting EEG signals that display structured spatiotemporal patterns across multiple electrodes. In EEG applications, CNNs are often applied to two- or three-dimensional data representations. A common approach encodes EEG recordings as time × channel matrices, enabling two-dimensional convolutions to extract temporal and spatial features concurrently (Rajwal and Aggarwal, 2023).

In seizure detection, they have been applied to both raw EEG recordings and spectrogram representations, achieving high accuracy in identifying epileptic patterns (Acharya et al., 2018). For sleep stage classification, CNN-based models outperform traditional methods by capturing frequency-specific components alongside temporal dynamics (Supratak et al., 2017). In areas such as mental workload estimation and emotion recognition, CNNs enable the automatic extraction of discriminative features from complex EEG signals, reducing reliance on manual feature engineering (Zhong et al., 2020). In neurological disorder detection, numerous studies have applied CNNs to epilepsy diagnosis using EEG data. Acharya et al. developed a 13-layer CNN that achieved an accuracy of 88.67% on the Bonn University database, demonstrating the feasibility of deep architectures for seizure detection without manual feature extraction (Acharya et al., 2018). These results highlight the potential of CNNs to deliver reliable, automated diagnostic support in epilepsy monitoring. CNNs have also been extensively employed in sleep stage classification, an important area in healthcare. Supratak et al. introduced DeepSleepNet, a hybrid model combining CNNs and bidirectional LSTM for automatic sleep staging from raw EEG signals, achieving 82% accuracy on the Sleep-European Data Format (EDF) dataset and demonstrating the ability of CNNs to capture both localized features and temporal dependencies (Supratak et al., 2017). More recently, Mousavi et al. improved CNNs architectures by integrating attention mechanisms, leading to enhanced classification accuracy across multiple sleep stage datasets (Mousavi et al., 2019).

Beyond their applications in neurological disorder detection and sleep analysis, CNNs have also been applied to mental workload estimation, motor imagery classification for rehabilitation, and BCI systems supporting assistive technologies. Lawhern et al. (2018) proposed EEGNet, a compact CNNs architecture specifically tailored for BCI applications, which demonstrated strong performance across several EEG paradigms, including motor imagery, P300, and error-related potentials (Lawhern et al., 2018). Beyond EEGNet, researchers increasingly tailor architectures for wearables and edge devices, emphasizing low power, small memory footprints, and robustness under reduced electrodes. Strategies include depthwise/group convolutions and bottlenecks, model compression (pruning, low-rank factorization, and 8−/4-bit quantization), and spatial simplification via dual- or few-channel montages. Increasingly, end-to-end channel selection (attention weights, sparsity penalties, or differentiable masks) learns which electrodes to retain while maintaining accuracy, enabling lean decoders suitable for real-time BCI and rehabilitation. For reproducibility and deployment relevance, studies should report params, model size and precision, hardware latency/energy, electrode montage/count, and performance with vs. without channel reduction/ compression (Wu et al., 2025; Zhao et al., 2022; Schneider et al., 2020). Despite these advantages, CNNs have notable limitations. Effective training typically demands large volumes of labeled data, which can be difficult to obtain in clinical EEG studies due to the cost and variability of manual annotation. Moreover, while CNNs excel at extracting spatial features, their capacity to capture complex temporal dependencies is limited unless combined with recurrent layers or other temporal modeling approaches.

### 4.2 RNNs and LSTM for long-term dependency processing

RNNs are a neural architecture designed to capture temporal dependencies in sequential data. To address the problem of modeling correlations across time steps, given an input sequence *x_1_, x_2_, …, x_T_*, the hidden state update in Equation (5) is defined as:


$$h_{t}=\sigma (W_{\mathrm{xh}}x_{t}+W_{\mathrm{hh}}h_{t-1}+b_{h})$$ (5)

where *h_t_* is the hidden state at time t, *W_xh_* and *W_hh_* are the input-to-hidden and hidden-to-hidden weights, respectively, and *b_h_* is the bias term. However, conventional RNNs encounter optimization instability due to gradient vanishing when processing long sequences.

LSTM is an improved form of RNNs. LSTM introduces memory blocks instead of conventional simple RNN units to handle the problem of vanishing and exploding gradient. LSTMs can handle long term dependencies much better than the traditional RNNs. This means that LSTMs can remember and connect previous information to the present. The LSTM memory cell consists of three gates: the forget gate, the input gate, and the output gate (Sharma et al., 2023).

The *C_t-1_* denote the cell state at time t-1, *h_t-1_* denote the hidden output at time t-1, and *x_t_* denote the current input at time t. The forget gate determines how much information from the previous cell state *C_t-1_* is retained. The output of the forget gate in Equation (6) is obtained as:


$$f_{t}=\sigma (W_{f}\cdot [h_{t-1},x_{t}]+b_{f})$$ (6)

where *σ* is the activation function, *W_f_* is the weight matrix of the forget gate, and *b_f_* is the bias term. The value range of *f_t_* is (0,1). The input gate controls how much new information will be written to the cell state. The gate output and the candidate cell content in Equations (7) and (8)are calculated as:


$$i_{t}=\sigma (W_{i}\cdot [h_{t-1},x_{t}]+b_{i})$$ (7)

$${\tilde{c}}_{t}=\tanh(W_{c}\cdot [h_{t-1},x_{t}]+b_{c})$$ (8)

where *W_i_* and *W_c_* are the weight matrices of the input gate and candidate state, *b_i_* and *b_c_* are the corresponding bias terms. The value range of *i_t_* is (0,1), while the value of *c_t_* is in (−1,1). The updated cell state in Equation (9) is computed by combining retained memory and new information:


$$c_{t}=f_{t}\cdot c_{t-1}+i_{t}\cdot {\tilde{c}}_{t}$$ (9)

The output gate determines how much of the cell state contributes to the final output in Equations (10) and (11) is defined as:


$$o_{t}=\sigma (W_{o}\cdot [h_{t-1},x_{t}]+b_{o})$$ (10)

$$h_{t}=o_{t}\cdot \tanh(c_{t})$$ (11)

where *W_o_* and *b_o_* represent the weight and bias of the output gate.

RNNs and LSTM in particular, have been demonstrated to be powerful for modelling the temporal dynamics in EEG signals. Their memory and processing of information from previous time steps, further makes them suitable for computer-aided applications where EEG montage proceeds in an ongoing manner (e.g., seizure prediction, emotion recognition, and mental workload estimation). Recent work has found using LSTM networks produce high detection accuracy, when generalizing across subjects. For instance, Roy et al. (2019) proposed a combined CNN–LSTM model for epilepsy seizure classification on the Temple University Hospital (TUH) dataset with accuracy over 93%. Likewise, Acharya et al. (2018) introduced an LSTM model for automatic diagnosis of epilepsy using EEG signals, the work showed the ability of the network to learn spatial and temporal features with raw signals without any handcrafted features involved. Researches based-LSTM have also made outstanding contributions in progress made in emotion recognition. Tripathi et al. (2017) deployed a deep LSTM model on the database for Emotion Analysis using Physiological signals in order to model long-term dependencies in affective EEG responses, and observed large gains over standard classifiers. Another area of application of interest is sleep stage classification. Mousavi et al. (2019) utilized an LSTM-based model to classify the five sleep stages with EEG data for the Sleep-EDF and reported state-of-the-art performance by capturing the complex temporal transitions between sleep cycles.

Moreover, lighter versions of LSTMs, the gated recurrent units (GRUs) have also been scrutinized for mitigation of the increasing LSTMs complexity for similar classification accuracy. However, there are still some problems to be solved as well. In addition, they are notoriously sensitive to class imbalances/column-row class distributions and strong labelled data, which are burdensome in diverse domains (Alqudah and Moussavi, 2025). Furthermore, for better interpretable model and an unsupervised domain adaptation problem that is applicable to different subjects, there is still a long way to go.

### 4.3 Transformer-based models

The Transformer model structure has also recently drawn a lot of attention in the field of EEG. Because it models long-range dependencies, it is highly parallelizable, and is interpretable because of the attention mechanisms. Self-attention is flexible to identify salient temporal and spatial features, without having to make any *a priori* attempts to determine which segments of the EEG are most relevant. For an input feature sequence *H_t_∈R*^*T* × *d*^, where *T* is the sequence length, *d* is the feature dimension, three learnable linear transformations are applied to generate the query, key, and value matrices, denoted as *Q, K*, and *V*, respectively in Equation (12) is defined as:


$$Q=H_{t}W_{Q},K=H_{t}W_{K},V=H_{t}W_{V}$$ (12)

where W_Q_, W_K_, W_V_∈R^d × d^ are the corresponding projection matrices. This operation enables each token to interact with others through the subsequent attention computation. The self-attention mechanism calculates a similarity score between the query and key matrices, which is then normalized to obtain the attention weight. The scaled dot-product attention in Equation (13) is defined as:


$$\text{Attention}\;(Q,K,V)=\text{Softmax}(\frac{{\mathrm{QK}}^{T}}{\sqrt{d}})V$$ (13)

where $\;\sqrt{d}$ is the scaling factor to prevent the dot product from growing too large when the dimension *d* increases, and Softmax normalizes the weights along each query dimension. The resulting matrix represents a weighted combination of all input positions, allowing the model to selectively focus on relevant tokens across the entire sequence. To capture diverse dependency patterns from different representation subspaces, multiple attention heads are computed in parallel. The outputs of these heads are concatenated and linearly transformed in Equation (14) is defined as:


$$\text{MultiHead}(H_{t})=\text{Concat}(A_{1},A_{2},\ldots ,A_{m})W_{O}$$ (14)

where *A_i_ = Attention (Q_i_, K_i_, V_i_)*, denotes the output of the *i*-th attention head, *m* is the total number of heads, and *W_O_∈R*^*md* × *d*^ is the output projection matrix. This multi-head mechanism enhances the model’s ability to jointly attend to information from different representation subspaces at different positions. To stabilize training and maintain gradient flow, a residual connection followed by layer normalization is applied to the output of the multi-head attention in Equation (15) is defined as:


$$H_{t}^{'}=\text{LayerNorm}(H_{t}+\text{MultiHead}(H_{t}))$$ (15)

where *H_t_* represents the input of the current encoder block, and *H*^***’***^*_t_* denotes the normalized output after the attention operation. Each position in the sequence is then independently processed by a fully connected feed-forward network to introduce nonlinearity and enhance feature transformation capability. The computation in Equation (16) is formulated as:


$$\mathrm{FFN}(x)=\text{ReLU}({\mathrm{xW}}_{1}+b_{1})W_{2}+b_{2}$$ (16)

where *W_1_, W_2_* are the weight matrices, and *b_1_, b_2_* are the bias terms. The RELU activation function introduces nonlinearity, enabling the network to learn complex mappings between input and output features. The output of the feed-forward network is again combined with the input via residual connection and layer normalization to form the final encoder output in Equation (17) is defined as:


$$H_{\text{out}}=\text{LayerNorm}(H_{t}^{'}+\mathrm{FFN}(H_{t}^{'}))$$ (17)

This ensures that the Transformer encoder effectively integrates both global contextual attention and local feature transformation, maintaining training stability and representational diversity.

Recently a number of transformer-based models have been proposed. It is intended to cover broad EEG applications with improved performance. For example, the Patched Brain Transformer applies the transformer architecture on the EEG data by dividing the spatial regions of the brain into patches. This architecture avoids the need for a fixed number of EEG channels and recording lengths, thus allowing for effective pre-training on diverse datasets (Zhang et al., 2023; Klein et al., 2025). Another remarkable method is Spectral Transformer, which first obtains the power spectral density estimates of EEG signals to the frequency domain and then performs transformer layers to capture frequency specific information. With deep ensemble learning, this method achieved classification accuracies of 96.1, 94.2, and 93.6% for the ensemble, temporal transformer, and spectral transformer models, respectively (Zeynali et al., 2023). For motor imagery classification, the Convolutional Transformer Network (CTNet) model combines a convolutional module that extracts local and spatial features from EEG time series with a transformer encoder to model high-level global correlations (Zhao et al., 2024). EEG model conformer bridges the gap between CNN and transformer by integrating convolution and transformer layers into a unified architecture, which can extract the local information and capture global information in the EEG signals, and achieves state-of-the-art results of EEG classification (Song et al., 2022). Meanwhile, the EEG-Patch Former is proposed for the attentive state decoding, and it integrates time series CNNs modelling frequency-based features with spatial–temporal patching modules in a transformer architecture. This architecture enables the representation to be learned jointly for both spatial and temporal information and contributes to better accuracy in decoding (Ding et al., 2025). Collectively, these studies draw attention to the flexibility and performance of transformer-based models for EEG decoding. They also show the feasibility of these methods to improve performance on a variety of EEG tasks.

Foundational models’ approach is being widely used by researchers to overcome, the high cost of expert annotation for clinical EEG data and the substantial variability inherent in neural signals. The core principle underlying EEG foundation models is that by learning rich, transferable representations from large amounts of unlabeled or weakly-labeled EEG data, models can capture universal neural dynamics that generalize across subjects, sessions, recording conditions, and even different electrode montages. Foundation models represent a paradigm shift in deep learning, where large-scale models are pretrained on massive datasets using self-supervised objectives and subsequently adapted to diverse downstream tasks through fine-tuning or prompting. Inspired by the success of foundation models in natural language processing (e.g., BERT, GPT) and computer vision (e.g., CLIP, MAE), the EEG research community has begun developing analogous approaches tailored to neurophysiological signals. These models address fundamental challenges in EEG analysis, including limited labeled data, high inter-subject variability, and the need for cross-dataset generalization.

### 4.4 Hybrid architectures for joint spatial–temporal representation learning

Hybrid DL architectures combining CNNs with RNNs, particularly LSTM networks, and more recently CNN-Transformer hybrids, have become very popular in EEG-related studies. These approaches are developed to take advantage of the best of each block. CNNs are good at learning spatial and the frequency-domain salient features of EEG, while LSTMs or Transformers are capable of learning the temporal relationships and long-range context with a sequence of EEG signals. Several previous works have demonstrated that CNN-LSTM can learn well the spatiotemporal patterns in EEG, and achieve better classification performance in applications such as epileptic seizure detection, sleep stage classification, and motor imagery recognition. For example, Roy et al. (2019) developed a CNN-LSTM model for seizure detection, in which convolutional layers were used to learn spatial features, while LSTM layers were employed to model temporal properties. This method resulted in clear enhancements as compared to conventional processing. Similarly, Mousavi et al. (2019) proposed a CNN-LSTM architecture for automated sleep staging with improved accuracy, which was claimed to be due to the joint modelling of spectral–spatial and temporal features in EEG. These results provide evidence that hybrid models may be useful to improve performance in a diverse set of EEG tasks.

Lately, Transformer-CNN hybrids have been introduced as a remedy for these limitations in RNN-based architectures, namely the slow training pace and incapability of capturing long-distance dependencies. In the following models, Transformer modules are formulated together with CNN feature extractors to exploit local spatial features and global contextual information. Yao et al. (2024) proposed a Transformer-CNN model for emotion recognition using EEG. In their architecture, local features were obtained from raw EEG signals using CNN layers, and temporal dependencies were learned by a Transformer encoder. This method achieved better performance than CNN and LSTM alone, especially on noisy EEG data and complex emotions, and proved the validity of the model. Similarly, Wu et al. (2022) proposed a hybrid model combining CNN with multi-head self-attention for motor imagery classification. Their method was more robust and generalizable across subjects than did the conventional CNN or RNN methods.

### 4.5 Graph neural networks (GNNs) for EEG channel modeling

Graph neural networks (GNNs) have emerged as a powerful paradigm for modeling EEG data by explicitly representing the brain’s electrode configuration as a graph structure G = (V, E, A), where V denotes the set of nodes (electrodes), E represents the edges (inter-electrode connections), and A ∈ ℝ^N × N^ is the weighted adjacency matrix encoding connection strengths between N electrodes. Unlike conventional deep learning architectures that treat EEG channels as independent or impose rigid grid structures, GNNs respect the intrinsic topology of the electrode montage and can capture complex functional and anatomical relationships that underlie neural dynamics providing an effective framework for modelling non-Euclidean structure especially promising for EEG, where electrodes form a natural graph and inter-channel connectivity carries task-relevant information (Graña and Morais-Quilez, 2023).

In EEG, edges can be defined in several principled ways: (i) geometric/anatomical (connect spatially proximate electrodes on the 10–20/10–10 layout; distance-weighted); (ii) functional connectivity (undirected weights from correlation, coherence, or band-limited phase locking value (PLV) within analysis windows); (iii) effective/directional coupling (directed links from Granger causality, partial directed coherence (PDC), or related measures); and (iv) learned/adaptive adjacency (attention-based or parameterized affinity, typically with sparsity/symmetry constraints). Many studies further adopt multi-graph/multi-band designs to fuse complementary priors. There have been some successful studies applying GNNs to EEG data for seizure detection, cognitive workload estimation and emotion recognition. For example, Xu et al. (2024) developed a spatial–temporal Graph Convolutional Network (GCN) and Bidirectional Gated Recurrent Unit (BiGRU) model for epileptic seizure recognition. Within this framework, one can model the topological structure of EEG channels as a network whose nodes correspond to channels, and edges represent either physical or functional links. The model captures spatial dependencies using graph convolution and temporal dynamics using recurrent units and achieves better performance compared to traditional CNN and RNN models. Similarly, Hou et al. (2022) proposed a GCN model for motor imagery classification by using the adaptive graph construction. Such a strategy allows the model to adaptively learn the subject-level inter-channel relationships and further deal with the quality control across different subjects. Our subject-adaptive graph models also substantially outperformed fixed graph structures on classification accuracy.

The construction of the adjacency matrix A is a critical design choice that significantly influences model performance. Given electrode coordinates p_i_ and p_j_, the adjacency weight in Equation (18) is computed as:


Aijdist=exp(−‖pi−pj‖2σ2) (18)

where σ is a bandwidth parameter controlling the decay rate of edge weights with distance.

In functional connectivity adjacency, edges are defined based on statistical dependencies between electrode signals. Common measures include Pearson correlation, coherence, and PLV. For band-limited PLV, the adjacency in Equation (19) is computed as:


$$A_{\mathrm{ij}}^{\mathrm{PLV}}=|\exp\;{\left(j(\varphi_{i}(t)-\varphi_{j}(t))\right)}_{t}|$$ (19)

where $\varphi_{i}(t)$ represents the instantaneous phase of the signal at electrode i, extracted via Hilbert transform, and (.)t denotes temporal averaging.

Besides spatial modelling, representation of graphs in the frequency domain has also been investigated. Li et al. (2022) introduced a spectral graph convolutional network on EEG signals in frequency domain. Spectral-spatial approach at the same time consider spectral and spatial information and have shown the good performance of sleep stage classification. Hybrid models which integrate GNN and attention mechanisms have also demonstrated the benefit of improving their model accuracy and interpretability. For example, Chen et al. (2024) used channel-wise attention combined with graph convolution layers to weight on EEG electrodes by task relevance during emotion recognition. This approach yielded not only better perfect now just correlation, but also localised the contributions to particular areas of the brain.

### 4.6 Transfer learning-based pre-trained knowledge generalization

Transfer and adaptation have been playing crucial roles in the field of EEG-based applications to cope with subject, session, and recording condition variability. The signatures of EEG signals exhibit high inter-subject variability, since they are influenced by individual anatomical, physiological, and psychological factors. This difference typically limits the applicability of models trained on one individual or on specific data. It has been widely recognized as a promising way to leverage learned information of some source domain/dataset for achieving favourable performance in the target domain when only small amount labeled data is available for the target domain, hence avoiding learning everything from scratch. Domain adaptation as a special case of transfer learning aims to minimize the distribution difference between source and target domains. This promotes reliable decoding of EEG across subjects and recording context.

Related works have employed transfer learning methods including finetuning pre-trained models, domain adaptation, or adversarial training for EEG classification. EEG-Former pretrains Transformers on compound datasets to yield universal, interpretable representations with strong cross-task transferability. Large-scale masked/contrastive pretraining has also produced the Large Brain Model for BCI, which scales to hundreds of millions of parameters and fine-tunes across classification and regression tasks with state-of-the-art results (Chen et al., 2024). Building on Biosignal Embedded Downsampled Representation (BENDR)‘s early evidence for device- and subject-invariant features, newer compact Transformers such as EEGPT demonstrate robust, few-label adaptation across datasets (Kostas et al., 2021). In parallel, modern domain-adversarial pipelines (dynamic source selection, cross-attention alignment) reliably improve cross-subject generalization for emotion and workload decoding, complementing finetuning with explicit distribution alignment. Taken together, these trends suggest a practical recipe: (i) self-supervised pretraining on multi-center EEG, (ii) lightweight subject-wise finetuning, and (iii) adversarial/contrastive alignment for residual shift—substantially reducing per-patient calibration time while increasing robustness in real-world BCI and clinical settings. For example, Liu et al. proposed a domain-adversarial neural network that learns domain-invariant EEG features, improving cross-subject motor-imagery classification accuracy (Liu et al., 2022). Other methods, such as Domain Adaptation, involves learning to align the feature distributions, without manual annotation for target data. In addition, there have been some recent attempts that embed transfer learning into deep architectures such as CNN, LSTMs and transformers, which can preserve more general features for adapting domain shifts. For instance, Li et al. (2024) presented a transformer-based domain adaptation model for EEG-based cognitive workload estimation by leveraging attention mechanisms to emphasise domain-invariant representations. These approaches increase the generalization of the model and shorten the calibration time which is crucial for the feasibility and user-friendliness of the EEG-based BCI. Much has been accomplished, there are still many obstacles. This negative transfer inflates when the source and target domains have significant difference, thus affecting the performance of model. On the other hand, there is no common set of measures to reliably detect domain shifts. To deal with these challenges, various current research topics are investigating more sophisticated adaptation techniques, multi-source domain adaptation, and personalized calibration for improving the robustness and scalability of EEG-based healthcare systems.

### 4.7 Self-supervised and contrastive learning

Self-supervised learning (SSL) pretrains EEG encoders on large unlabeled corpora via proxy objectives, then adapts them with scarce labels for downstream tasks (sleep staging, seizure detection, affect, motor imagery (MI)-BCI). Early clinical studies show that SSL approaches can match or surpass fully supervised baselines under low-label regimes while improving cross-subject transfer (Banville et al., 2021). Contrastive SSL builds subject-robust embeddings by maximizing agreement between two augmented views of the same segment and pushing apart negatives; in EEG, effective views include temporal cropping, time–frequency transforms, channel dropout/perturbation, and cross-session pairing. Generic time-series methods (e.g., temporal–contextual contrasting) have been adopted with open implementations, facilitating reuse and comparison. Large-scale EEG-specific contrastive pretraining (e.g., BENDR-style Transformers) further supports “thinker-invariant” representations that adapt across devices and tasks and consistently improve few-label performance on public benchmarks (e.g., Sleep-EDF) (Kostas et al., 2021). Masked/reconstruction SSL complements contrastive learning by reconstructing withheld time or time–frequency patches to bias encoders toward local spectral–temporal structure; recent variants extend masking to graph-structured EEG over electrode montages, aiding transfer between high- and low-density systems and enabling distillation. Backbones are agnostic, CNNs, RNN, Transformers, and GNNs, typically combined with DL-based denoising and evaluated via subject-wise cross-validation within reproducible ecosystems (MOABB; Braindecode/MNE-Python) (Weng et al., 2025; Borra et al., 2024). Collectively, SSL reduces calibration burden and enhances robustness when labels are limited, provided that augmentations are physiologically plausible and data-leakage safeguards are enforced.

## 5 Healthcare applications using EEG-based deep learning

DL has led to many applications in healthcare, where EEG signals are used to decode neurological, cognitive, and emotional states with high accuracy. Unlike traditional approaches, DL models can automatically learn discriminative spatiotemporal patterns, providing reliable and scalable solutions across different clinical domains. Key applications include neurological disorder diagnosis, BCIs, sleep and fatigue monitoring, mental health assessment, and neurorehabilitation. To give a structured overview, Table 3 summarizes these domains together with their representative EEG paradigms, commonly used deep learning architectures, main outcomes, and widely adopted benchmark datasets. This structured summary highlights both the breadth and translational potential of EEG-based DL in healthcare. The following subsections (5.1–5.5) elaborate on each application domain in greater detail, with emphasis on methodological advances, performance trends, and clinical implications. We evaluate models with the confusion matrix. For a binary decision, let TP (true positives), TN (true negatives), FP (false positives), and FN (false negatives) denote the cell counts; for multi-class tasks, we compute these per class in a one-vs-rest manner and aggregate (macro/micro). Formal definitions include Accuracy = (TP + TN) / (TP + TN + FP + FN); Sensitivity/Recall = TP / (TP + FN); Specificity = TN / (TN + FP); Precision = TP / (TP + FP); F1 = 2·(Precision·Recall)/ (Precision + Recall). Although different applications may prioritize different operating points, we report and compare models primarily by Accuracy because it is a universal, task-agnostic summary across Section 5 scenarios (e.g., seizure detection, sleep staging, affect recognition).

**Table 3:** EEG healthcare applications enabled by deep learning.

Category	Application domain	EEG paradigm	Deep learning models	Key outcomes	Example datasets
Clinical Diagnosis	Neurological Disorder Diagnosis (Epilepsy, AD, PD)	Resting EEG, Task EEG	CNN, LSTM, CNN–LSTM, Transformer, GNN	>90% accuracy in seizure detection; early biomarkers for AD/PD identified	Bonn, CHB-MIT
Functional Monitoring	Brain–Computer Interfaces (BCIs)	Motor Imagery, SSVEP, P300	CNN, CNN–LSTM, Transformer	High information transfer rate (ITR); real-time decoding; robust cross-subject performance	BCI Competition IV
	Sleep & Fatigue Monitoring	Polysomnography EEG	CNN, SeqSleepNet, Transformer	Automated sleep staging (>85% accuracy); real-time fatigue detection	Sleep-EDF
Mental Health	Emotion & Mental Disorder Assessment	Resting/Task EEG	CNN, LSTM, Transformer, GNN	High accuracy in depression and emotion detection; interpretable biomarkers for psychiatric disorders	DEAP, SEED
Rehabilitation & Therapy	Motor Rehabilitation & Neurofeedback	Motor Imagery EEG	CNN, RNN, GCN, Attention-enhanced Transformers	Improved motor recovery in stroke; adaptive neurofeedback enhances cognitive performance	Custom clinical datasets

### 5.1 Neurological disorder diagnosis

Epilepsy is a neurological disorder characterized by recurrent seizures. It is also one of the most extensively studied conditions using EEG in combination with DL. Early seizure detection is critical to mitigate adverse effects and improve patient outcomes. CNNs have been widely adopted for seizure detection, extracting spatial–temporal features directly from raw EEG data. CNNs learn local spatial-spectral motifs from EEG, but generalization and false-alarm rates remain challenging in unattended settings. Acharya et al. (2018) proposed a CNNs model that outperformed traditional classifiers in seizure detection, while Truong et al. (2018) demonstrated that CNNs combined with LSTM layers effectively capture temporal dependencies, enabling seizure prediction. More recently, Roy et al. (2019) applied attention-based deep learning frameworks for robust seizure detection, enhancing sensitivity and specificity across diverse datasets. For epilepsy, clinically useful detectors must pair very high sensitivity with very low false alarms. The International League Against Epilepsy (ILAE) and International Federation of Clinical Neurophysiology (IFCN)‘s validation standard for ambulatory devices requires ≥90% sensitivity and reports false-alarm rates ≈0.2–0.67 per 24 h in phase-3 studies of convulsive seizures—illustrating the practical ceiling patients and caregivers can tolerate; reports must also include latency and device-deficiency time. Simulation work on real-world use cases confirms that sensitivity drives clinical benefit, while False Alarm Rate (FAR) must remain low (generally ≤ a few alarms/day) to maintain utility in trials and home monitoring (Goldenholz et al., 2024). By contrast, many recent EEG-Artificial Intelligence (AI) papers still show False Discovery Rate (FDR) around 0.5/h or FAR≈0.1–0.2/h—insufficient for unattended use without human triage—underscoring the need for personalization and external validation (Wang and Jing, 2025). AD, a progressive neurodegenerative disorder, presents characteristic EEG alterations such as slowed rhythms and decreased signal complexity. Early diagnosis is challenging due to subtle EEG changes at initial stages. Hybrid CNN-LSTM architectures have been employed to model both spatial and temporal EEG features indicative of AD. Hybrids CNN-LSTM capture temporal dynamics and early trajectories, at the cost of parameters, tuning, and latency. Kowshiga et al. (2024) reported effective early-stage AD detection using such hybrids, while recent studies by Abadal et al. (2025) integrated graph neural networks with DL to capture the brain’s network-level alterations in AD patients. Transformer-based models have also been explored for their ability to focus on salient EEG features related to cognitive decline, improving classification performance. Transfer learning aids cross-site generalization but risks negative transfer, requiring calibration. For AD, EEG-DL tools become clinically relevant when they demonstrate multi-site, external validation with area under the receiver-operating characteristic curve (AUROC) ≥ 0.85 and robustness to montage/age/medication, ideally leveraging network-level GNN features that capture connectivity changes in early disease. Recent reviews and GNN studies support this direction but emphasize standardization and head-to-head testing (Toumaj et al., 2024). GNNs encode connectivity and offer interpretability, but are sensitive to graph/montage design and small cohorts. PD affects motor function and is associated with altered EEG rhythms, including beta and alpha band abnormalities. DL approaches have shown promise in early PD diagnosis and symptom monitoring by capturing these EEG biomarkers. Oh et al. (2020) developed CNN-based models for PD detection with high accuracy, while Arasteh et al. (2021) utilized transfer learning to improve model generalizability across EEG datasets. Additionally, multimodal DL frameworks combining EEG with other biosignals have demonstrated enhanced PD classification capabilities. Multimodal fusion yields further gains with integration complexity. Translation demands external multi-site validation, operating-point reporting, low FAR for epilepsy, and channel-minimal, robust pipelines. As for PD, promising EEG pipelines (channel-attention CNNs, time-frequency transforms, and hybrid models) report strong cross-subject results; translation will require external cohorts, salient operating-point reporting (AUROC/sensitivity at fixed specificity), and channel-minimal designs for routine clinics (Mukherjee and Roy, 2025).

### 5.2 Brain-computer interfaces (BCIs)

BCIs offer a promising pathway in healthcare, particularly for restoring communication and control in individuals with severe motor impairments. By decoding neural signals, most commonly obtained through EEG, into executable commands, BCIs enable interaction with external devices such as wheelchairs, robotic manipulators, and communication systems. Noninvasive healthcare BCIs must balance information transfer rate (ITR) with calibration burden and user comfort. These technologies hold the potential to restore functional independence and substantially improve quality of life for patients affected by spinal cord injury, stroke, amyotrophic lateral sclerosis (ALS), and other neurodegenerative conditions.

Among EEG-based paradigms for BCIs, MI, Steady-State Visual Evoked Potentials (SSVEP), and P300 event-related potentials are the most widely used. Their popularity comes from their distinct neural signatures and proven effectiveness. MI involves the imagination of specific motor actions, such as hand or foot movements, which produce changes in sensorimotor rhythms detectable in EEG signals. These patterns can be decoded in real time to control external devices, including wheelchairs and prosthetic limbs. DL models, particularly CNNs and LSTMs, have significantly improved MI classification accuracy by capturing both spatial and temporal EEG features (Roy et al., 2019). Despite requiring user training and sustained mental effort, MI BCIs remain popular due to their direct link to voluntary motor control. For MI, CNN/Transformer stacks capture sensorimotor rhythms and non-stationary dynamics, boosting cross-session decoding; yet subject drift, heavy calibration, and latency under tight real-time budgets persist. SSVEP-based BCIs use visual stimuli flickering at specific frequencies to elicit steady-state responses in the visual cortex, which can be detected reliably in EEG recordings. These responses serve as markers for selecting commands in BCI applications. SSVEP benefits from compact CNNs that learn harmonic/phase structure and deliver high ITR with short calibration; limits include visual fatigue, display-hardware latency/phase jitter, and diminished performance under low-contrast or dry-electrode setups. SSVEP systems are favored for their high information transfer rates and relatively short calibration times. However, prolonged exposure to flickering stimuli can cause user discomfort and fatigue. Recent advances in DL have enhanced SSVEP decoding by automatically extracting spectral features, enabling more robust and efficient classification (Chen et al., 2023). The P300 paradigm exploits event-related potentials elicited approximately 300 milliseconds after an infrequent or unexpected stimulus. P300 gains from attention and ensembles that enhance low-SNR target detection; however, severe class imbalance, variable latencies, and susceptibility to ocular/muscle artifacts remain. P300 BCIs typically employ an oddball paradigm where the target stimulus is embedded among frequent non-target stimuli, and the presence of P300 signals indicates the user’s intention. This paradigm is widely used in spellers and device control, valued for its minimal training requirements and ease of use. However, the P300 signal’s low signal-to-noise ratio and susceptibility to artifacts pose challenges for accurate detection. DL approaches integrating attention mechanisms and ensemble learning have demonstrated improved P300 detection performance (Daǧ et al., 2022).

Successful deployment of non-invasive EEG BCIs in healthcare hinges on two user-facing constraints: ITR sufficient for fluid interaction and calibration time short enough for routine clinical or home use. Among established paradigms, SSVEP offers the most favorable trade-off. With frequency/harmonic filter banks (or attention over harmonics), ergonomic stimulus layouts, and a few per-class samples, contemporary systems routinely achieve comfortable throughput with ≤2–10 min calibration, making them strong candidates for text entry and discrete device selection. Visual fatigue remains the key design constraint. P300 interfaces provide low training burden with moderate ITR. Robustness in real-world use depends on class-imbalance handling, adaptive stopping, and multi-trial aggregation to stabilize low-SNR ERPs. P300 suits users who cannot sustain steady gaze on flicker stimuli or when rapid onboarding is paramount (Bai et al., 2023). MI affords natural, volitional control, but throughput is lower and calibration is longer. Translation is improving through self-supervised pretraining on large unlabeled EEG, parameter-efficient personalization (e.g., adapters, prototype layers, batch-norm tuning), and online co-adaptation that updates the decoder during use (Pan et al., 2025). These strategies reduce subject-specific data demands and shorten time-to-control. Across paradigms, adoption is ultimately driven by human factors—comfort, setup time, and cognitive workload—more than peak accuracy. Clinically viable systems favor lightweight caps/electrodes, integrated artifact mitigation, and plug-and-play workflows. Explainability supports clinician trust; privacy-preserving learning facilitates multi-site training; and on-device inference lowers latency and infrastructure cost.

### 5.3 Sleep and fatigue monitoring

Sleep and fatigue monitoring through EEG analysis has become a pivotal area in healthcare due to its relevance in diagnosing sleep disorders and preventing fatigue-related accidents. EEG signals reflect dynamic brain activity patterns that vary distinctly across sleep stages, making them a primary modality for sleep stage classification and fatigue assessment.

Sleep staging, traditionally performed through manual scoring based on polysomnography, is time-consuming and susceptible to inter-rater variability. DL approaches, particularly CNNs and RNNs, have shown remarkable success in automating sleep stage classification from EEG. CNNs (incl. shallow) extract robust spectral–morphological cues from short epochs and run in real time, but miss long-range context and degrade with channel/montage shifts. RNN capture stage transitions and circadian dynamics, improving continuity scores, yet add latency and are sensitive to class imbalance. For instance, DeepSleepNet, a CNN-LSTM hybrid model, showed strong performance in classifying five sleep stages using raw single-channel EEG data without the need for hand-crafted features (Supratak et al., 2017). Similarly, SeqSleepNet used hierarchical recurrent layers and attention mechanisms to better capture temporal transitions between sleep stages (Phan et al., 2019). More recently, transformer-based models have improved temporal modeling further, allowing robust stage classification even when data are noisy or incomplete (Dai et al., 2023). Transformers excel at noisy/incomplete data via global attention and yield state-of-the-art on cross-night generalization, but require larger datasets, careful regularization, and more compute. Fatigue and drowsiness detection is also critical in areas such as driving safety and occupational health. EEG-based systems can track alertness in real time and detect early signs of fatigue before behavioral symptoms appear. Many real-time systems employ shallow CNNs or temporal models such as LSTMs to extract fatigue-related features from short EEG segments. In recent studies, transformer models have been employed to enhance the detection accuracy by modeling long-range dependencies in EEG sequences (Hassan et al., 2025). Despite these advancements, challenges remain, such as individual variability in EEG patterns and the influence of environmental factors. To address this, domain adaptation and personalized model fine-tuning have been introduced to improve model generalizability across subjects (Li et al., 2019). Additionally, multi-modal integration of EEG with EOGs and EMGs is being explored to enhance sleep and fatigue monitoring performance. Hybrids (CNN-LSTM/Transformer) offer best accuracy with tuning burden. Personalization/domain adaptation mitigates subject variability; multimodal EEG + EOG/EMG boosts REM/artefact robustness, at the cost of sensors and integration complexity.

For sleep staging, clinically deployable algorithms should perform at least within the human–human agreement envelope and report Cohen’s *κ* (alongside accuracy, macro-F1, and per-stage F1). A recent meta-analysis of manual scoring places expert inter-rater reliability around κ ≈ 0.76 (overall accuracy≈80%), providing a pragmatic non-inferiority threshold for automated systems. Accordingly, we recommend κ ≥ 0.76 with per-stage F1 ≥ 0.80 for N2/N3/REM and F1 ≥ 0.50 for N1, plus external validation across heterogeneous cohorts/scoring sites (Lee et al., 2022). Robust, multi-cohort systems such as U-Sleep have matched the best human experts on unseen clinics and shown resilience to montage variations, supporting readiness for workflow integration (single-EEG + EOG, laptop-CPU inference, and high-frequency outputs that can be aggregated to 30-s epochs). Subsequent work further confirmed U-Sleep’s robustness to American Academy of Sleep Medicine (AASM) montage recommendations, strengthening cross-site generalizability—critical for clinical adoption (Fiorillo et al., 2023). Recent prospective validations of deep models also report *κ* ≈ 0.82–0.85 against experts, exceeding human inter-rater medians (Cho et al., 2022).

For fatigue monitoring, clinical utility hinges on timeliness and generalization (cross-subject/setting) rather than headline accuracy alone. We recommend reporting AUROC under external validation, targeting AUROC≥0.85 with decision latency < 1 s using short windows (≈2–5 s) suitable for real-time alerts; models should also document channel count (prefer≤1–3 scalp channels for wearability) and fail-safe behavior. Contemporary reviews and cross-subject studies underscore EEG’ s sensitivity to early drowsiness, with recent Transformer/GNN pipelines showing improved cross-subject performance and efficient channelization for wearable deployment (Othmani et al., 2023). Early real-time and Transformer-based systems report strong accuracy under realistic driving tasks, but emphasize the need for standardized endpoints and external testing before routine use (Hassan et al., 2025).

### 5.4 Mental health and emotion recognition

Mental health disorders such as depression, anxiety, and schizophrenia represent a major global health burden. Conventional diagnostic approaches often depend on subjective assessments, which may result in delayed or inaccurate diagnosis. EEG offers a non-invasive and objective method for evaluating brain activity related to mental states. In recent years, DL has been increasingly applied to EEG data to support early diagnosis, emotion recognition, and personalized monitoring of mental health.

Several studies have shown that EEG signals can be used to distinguish individuals with mental health conditions from healthy controls. For example, CNNs trained on resting-state EEG have been able to detect depression-related patterns with higher accuracy than traditional machine learning methods. RNNs, particularly LSTM models, are effective for capturing temporal dynamics in EEG and have been applied to monitor mood changes and detect anxiety episodes in real time. RNN capture mood dynamics and anxiety episodes but incur latency and suffer from class imbalance and drift. More recently, attention mechanisms and transformer-based architectures have been explored to improve interpretability and increase sensitivity to subtle brain activity changes linked to mental disorders (Konkar and Qu, 2025). Transformers/attention boost sensitivity to subtle, distributed patterns and offer saliency maps, but demand larger cohorts and careful regularization. \Emotion recognition from EEG has become an important area in affective computing, with applications in human–computer interaction, mental wellness tools, and therapeutic interventions. Emotional states are reflected in brain activity, particularly in the frontal and temporal regions. DL models have been trained on benchmark datasets such as Database for Emotion Analysis using Physiological signals (DEAP) and SJTU Emotion EEG Dataset (SEED) to classify either continuous valence*—*arousal dimensions or basic emotions including happiness, sadness, relaxation, and fear. CNNs are commonly used to learn spatial features from EEG channel maps, while LSTMs capture the temporal evolution of emotional states (Li et al., 2019). More recent approaches combine these models in hybrid frameworks or employ GNNs to model inter-channel connectivity (Lee et al., 2025). GNNs exploit connectivity for interpretability, with performance contingent on graph design. Transformer-based models have also demonstrated superior performance in capturing long-range dependencies across time and channels for affect recognition (Zhong et al., 2020). In addtion, emerging neurobiological models implicate the glymphatic system, a sleep-dependent waste-clearance pathway, in the pathophysiology of several psychiatric disorders via neuroinflammatory and metabolic mechanisms (Barlattani et al., 2024). This motivates EEG–clinical integration: prioritizing sleep-architecture biomarkers as proxies for glymphatic efficacy; pairing EEG with glymphatic-sensitive imaging to link electrophysiology with clearance dysfunction; and testing whether sleep-targeted therapies yield aligned improvements in EEG markers and symptoms. Such designs can foster mechanism-anchored biomarkers for early detection and treatment monitoring across depression, anxiety, and schizophrenia (Barlattani et al., 2025; Bersani et al., 2021).

EEG offers an objective window on mental states, and recent deep models are pushing it toward clinically useful screening and monitoring. For depression, multi-center reviews and new systems report competitive accuracy with compact CNN/Transformer pipelines and cloud/wearable deployment, suggesting feasibility for adjunctive diagnosis and longitudinal tracking (Elnaggar et al., 2025). For schizophrenia and related spectra, resting-state EEG with modern machine learning and DL yields robust differentiation, and recent studies even separate schizophrenia from bipolar disorder using spectral-entropy composites, which are an important step toward specificity (Rahul et al., 2024). For emotion/affect recognition, which underpins digital phenotyping and therapy feedback, state-of-the-art models increasingly couple CNN front-ends with Transformers or GNNs to capture long-range temporal dependencies and electrode-topology structure, achieving strong results on DEAP/SEED and newer cohorts (Elnaggar et al., 2025). Emerging dynamic graph attention formulations further improve cross-subject performance and interpretability by modeling time-varying connectivity. For clinical translation, we recommend: (i) external, site-held-out validation with effect sizes reported alongside accuracy/F1/AUROC; (ii) sensitivity analyses for medication state, comorbidity, and sleep; (iii) channel-minimal designs (≤4–8 electrodes) with on-device inference; and (iv) transparent explanations (saliency or graph attributions) aligned with neurophysiology. These steps, which are common in leading studies now, can convert promising lab-grade EEG biomarkers into scalable tools for screening, relapse detection, and treatment monitoring across depression, anxiety, and psychotic disorders (Ma et al., 2024).

### 5.5 Rehabilitation and neurofeedback

EEG-based DL approaches are gaining significant traction in rehabilitation, particularly for post-stroke motor recovery and cognitive neurofeedback applications. In post-stroke therapy, EEG provides real-time insight into neural activity that can be harnessed to track motor intention and brain reorganization. DL models have been employed to decode MI signals from stroke patients, enabling BCI-controlled rehabilitation devices. For example, Ma et al. (2024) proposed a hierarchical attention-based RNN to decode motor intention, allowing accurate control of upper-limb prosthetics in chronic stroke patients (Bi et al., 2021). Closed-loop neurofeedback systems, which provide real-time feedback or stimulation based on decoded neural signals, have also shown promise in accelerating neuroplasticity and recovery. These systems depend on precise temporal decoding of motor or cognitive states. Jin et al. (2024) developed a real-time closed-loop EEG-based BCI using deep RNNs, which improved motor scores in hemiparetic patients following a 6-week rehabilitation program. Another study by Tsuchimoto et al. (2019) used deep generative models to predict neural states and adapt neurofeedback tasks accordingly, improving attention and motor coordination in post-stroke subjects. Advanced models like GCN and attention-enhanced transformers have further improved the robustness of EEG decoding by learning dynamic spatial–temporal features across multiple brain regions. Hou et al. (2022) employed a GCN framework to analyze connectivity changes during rehabilitation and deliver neurofeedback that targeted disrupted motor networks, showing measurable gains in hand dexterity.

In addition to motor rehabilitation, EEG-based neurofeedback is also used to promote cognitive recovery. Transformer-based models have been trained on working memory and attention task EEG data to provide adaptive neurofeedback in patients with traumatic brain injury or cognitive decline, showing increased engagement and improved task performance (Zhang and Li, 2025). Multimodal approaches combining EEG with functional near-infrared spectroscopy (fNIRS) or EMG have been proposed for more comprehensive closed-loop systems. Moreover, unsupervised and self-supervised learning paradigms are being explored to improve generalization across subjects and sessions. N’dir et al. developed a contrastive learning framework for EEG-based neurofeedback, enabling zero-shot transfer to new participants (N'dir and Schirrmeister, 2025). These advances aim to address the current challenges of inter-subject variability and limited training data in clinical EEG applications.

EEG-driven BCIs and neurofeedback are advancing from proof-of-concept to clinically actionable tools for motor and cognitive rehabilitation. For post-stroke upper limb recovery, randomized trials now show that BCI-augmented therapy produces significant functional gains over dose-matched controls, strengthening the case for routine adjunctive use (Wang et al., 2024). Concurrently, online decoding has progressed to fine-grained motor commands (e.g., finger-level control), illustrating the headroom for richer, task-oriented training paradigms. On the algorithmic side, Transformers and graph neural networks improve robustness by modeling long-range temporal structure and inter-regional connectivity; recent clinical EEG studies demonstrate their utility for MI decoding and subject-shift resilience, a key barrier to at-scale deployment (Liu et al., 2024). Neurofeedback is likewise maturing: scoping reviews report consistent improvements in sensorimotor function with EEG-based protocols, while hybrid systems that couple EEG with fMRI show larger, targeted effects by aligning electrophysiology with hemodynamics to drive network-specific plasticity (Cioffi et al., 2024). Overall, the evidence base now supports BCI-assisted rehabilitation and targeted neurofeedback as credible complements to conventional therapy, provided systems meet real-world constraints on setup time, comfort, and longitudinal robustness (Wang et al., 2024).

## 6 Challenges and future directions

Table 4 summarizes the primary challenges encountered in applying DL to EEG-based healthcare, along with representative technical solutions and key references. The table condenses the issues discussed in Sections 6.1–6.6, providing a quick reference for researchers seeking to address limitations such as data scarcity, inter-subject variability, low signal-to-noise ratio, and model interpretability, as well as practical concerns in real-time deployment, multimodal integration, and regulatory compliance.

**Table 4:** Challenges and proposed solutions in EEG-based deep learning.

Challenge	Impact on EEG analysis	Proposed technical solutions
Data Scarcity	Limits model generalizability; hampers training of deep architectures.	Data augmentation (noise injection, time/frequency warping), synthetic data generation (GANs), transfer learning from large EEG/non-EEG datasets, multi-site data sharing initiatives.
Inter-/Intra-Subject Variability	Degraded cross-subject performance; increased calibration needs.	Domain adaptation, personalized models, subject-invariant feature learning, adaptive fine-tuning.
Low Signal-to-Noise Ratio (SNR)	Reduced reliability of detected neural patterns, especially in real-time applications.	Advanced artifact removal (ICA, wavelet), multimodal fusion (EEG + EMG/ECG), denoising autoencoders.
Model Interpretability	Limits clinical adoption due to “black-box” nature of deep models.	Explainable AI (LRP, Grad-CAM, SHAP), attention mechanisms, hybrid models combining interpretable features with DL outputs.
Real-Time Deployment	High latency, high computational load on edge devices.	Model compression (pruning, quantization, knowledge distillation), lightweight architectures (EEGNet), hardware–software co-design.
Multimodal Integration Challenges	Complexity in synchronizing heterogeneous signals; increased computational demands.	Joint spatial–temporal feature learning, cross-modal transformers, synchronized wearable sensor systems.
Regulatory and Ethical Issues	Delayed clinical approval; potential bias, privacy, and security concerns.	Compliance with FDA/EMA guidelines, diverse datasets for fairness, secure and anonymized data handling, informed consent.

### 6.1 Data scarcity, leakage and imbalance

One of the fundamental challenges in deploying deep learning techniques for EEG-based healthcare applications is the scarcity of large, high-quality, and labeled datasets. EEG data acquisition is a labor-intensive process that requires specialized equipment and clinical oversight, which limits the availability of diverse datasets. Furthermore, ethical and privacy constraints prevent sharing of medical EEG data between institutions, thus decreasing data availability even more (Roy et al., 2019). Publicly available EEG recordings typically suffer from limited sample size, short recording time, and biased demographics. These limitations limit the generalizability of trained models to larger populations (Aggarwal and Chugh, 2022). Another challenge is data leakage. It occurs when information from the test set inadvertently influences model training, leading to inflated performance estimates that fail to generalize. In EEG research, leakage commonly arises from: (i) overlapping epochs from the same recording session appearing in both training and test sets due to sliding-window segmentation; (ii) performing normalization, artifact rejection, or feature extraction on the entire dataset before splitting; (iii) hyperparameter tuning on test data rather than a held-out validation set; and (iv) subject-level leakage, where different segments from the same participant appear in both training and test sets despite subject-independent claims. Several studies reviewed here do not explicitly describe their data partitioning strategy, making it impossible to assess whether leakage occurred. Best practices require strict temporal and subject-level separation, with all preprocessing fitted only on training data. Researchers should explicitly report their cross-validation scheme, window overlap parameters, and whether any global statistics were computed before splitting (Roy et al., 2019).

EEG datasets are inherently imbalanced: seizures represent less than 1% of continuous recordings in epilepsy; sleep stage N1 is underrepresented; and target responses in P300 BCIs occur at roughly 1:6 ratios, representing a big challenge. Accuracy alone can be misleading in such contexts—a trivial classifier predicting only the majority class achieves high accuracy but zero clinical utility. Many reviewed studies report accuracy without complementary metrics such as sensitivity, specificity, precision, F1-score, or AUROC. Furthermore, class-rebalancing strategies (oversampling, SMOTE, class-weighted loss functions, or focal loss) are inconsistently applied and reported. Future studies should explicitly state the class distribution, justify the choice of evaluation metrics, and report per-class performance alongside aggregate measures.

### 6.2 Subject variability, dependent vs. independent evaluation

There is also a high inter- and intra-subject variability of EEG signals which is a significant drawback. This variability across recordings is influenced by factors including age, gender, cognitive state, electrode position and environmental noise. This variability makes it hard to transfer models trained on one cohort to another. Subject independent models typically suffer degraded performance because of this distributional shift. Given this variability, the studies distinguish subject-dependent (SD) from subject-independent (SI) evaluation. SD trains/tests within a single subject (typically across sessions) and often reports higher accuracy but limited translational value. SI targets generalization to unseen subjects, typically via leave-one-subject-out (LOSO)—training on N–1 subjects and testing on the held-out one. Given clinical and wearable deployment requirements, SI/LOSO should serve as the primary evaluation, with SD reported as a secondary upper bound (Roy et al., 2019). Several strategies have been proposed to mitigate these issues. Data augmentation techniques, such as signal transformation, noise injection, and synthetic data generation using GAN, aim to expand dataset diversity (Luo et al., 2020). Despite these advances, overcoming data scarcity and subject variability remains an open research problem that limits the real-world deployment of EEG-based DL systems. Large-scale, multi-site collaborations and standardized EEG benchmarks are urgently needed to promote reproducibility and robustness across diverse patient populations.

### 6.3 Model interpretability and explainability

Despite the impressive performance of DL models in EEG-based healthcare applications, their “black-box” nature remains a significant barrier to clinical adoption. Clinicians and healthcare practitioners require not only accurate predictions but also a clear understanding of how and why a model arrives at a specific decision. This is particularly important in high-stakes settings such as epilepsy detection or Alzheimer’s diagnosis, where interpretability could influence treatment decisions. However, the hierarchical and non-linear structure of deep neural networks makes it difficult to trace the influence of specific EEG features on the final output.

Recent studies have begun addressing this challenge by integrating explainable AI (XAI) frameworks into EEG analysis. Techniques such as layer-wise relevance propagation (LRP), GNNExplainer and shapley additive explanations (SHAP) have been employed to highlight informative EEG channels or time intervals that contribute most to a model’s prediction. For instance, Sturm et al. applied LRP to deep CNNs trained on EEG data to visualize decision-relevant brain regions, improving trust in automated seizure detection. Likewise, in GNN-based EEG pipelines, GNNExplainer provides local explanations by extracting predictive subgraphs and feature masks (e.g., frequency-specific edge weights), thereby identifying electrode pairs and connections that drive classifications and enabling principled channel/edge reduction without sacrificing performance (Zhdanov et al., 2022). Another notable trend is the use of attention mechanisms, not only to enhance performance but also to provide insight into which temporal or spatial features the model prioritizes during classification tasks (Roy et al., 2019). Moreover, inherently interpretable models, such as attention-augmented recurrent networks and sparse neural architectures, have been proposed to balance accuracy with transparency (Zeng et al., 2025). Despite these efforts, interpretability methods often face limitations, such as lack of consensus on what constitutes a “satisfactory” explanation, and their effectiveness can vary depending on the model type and application. Future research should emphasize the development of standard evaluation protocols for interpretability in EEG-based systems and design user-centered explanations that align with clinical reasoning. Only through such advancements can DL models gain the trust required for widespread integration into healthcare workflows.

### 6.4 Real-time deployment, edge computing and clinical generalizability

Real-time deployment of DL models for EEG-based healthcare applications presents significant technical challenges, primarily due to the high computational demands of complex neural networks and the need for low-latency processing. EEG signals are often streamed continuously and require rapid, accurate interpretation to enable timely interventions, such as seizure detection, neurofeedback, or assistive device control. Traditional cloud-based processing introduces latency and raises privacy concerns, making edge computing an attractive alternative that processes data locally on wearable or portable devices (Carvalho et al., 2021). However, implementing state-of-the-art DL models on resource-constrained hardware, such as microcontrollers or mobile System-on-Chip, demands careful model optimization and hardware-software co-design.

Recent advances in model compression techniques—including pruning, quantization, and knowledge distillation—have enabled the deployment of lightweight models suitable for edge devices without substantial loss of accuracy. For instance, EEGNet and its variants have been widely adopted for their compact architecture, balancing efficiency and performance for real-time EEG decoding (Lawhern et al., 2018). Moreover, novel neural network architectures, such as spiking neural networks and binarized neural networks, are gaining interest due to their potential for ultra-low-power operation compatible with embedded systems (Roy et al., 2019). Future research is focused on developing adaptive models that can efficiently update on-device, optimizing hardware accelerators tailored for EEG signal characteristics, and designing user-friendly wearable systems that integrate seamlessly into daily life (Karageorgos et al., 2020). These advances will be critical in translating DL-powered EEG healthcare solutions from laboratory prototypes into practical, widely accessible clinical tools.

Another challenge is related to clinical generalizability A model demonstrating high performance on a single benchmark dataset does not necessarily generalize to clinical populations. Factors limiting generalizability include: (i) acquisition heterogeneity—differences in EEG hardware, electrode placement, sampling rates, and referencing schemes across institutions; (ii) demographic confounds—training cohorts often lack diversity in age, sex, medication status, and comorbidities; (iii) dataset-specific artifacts—lab-based recordings may not reflect ambulatory or real-world noise conditions; and (iv) label quality—inter-rater variability in manual annotations (e.g., sleep staging, seizure marking) introduces systematic bias. Few studies in this review perform external validation on independent, multi-site datasets. For clinical translation, models should be tested across at least two independent cohorts, with explicit reporting of cohort characteristics, acquisition protocols, and performance stratified by relevant clinical variables.

### 6.5 Integration with multimodal biosignals (EMG, ECG, etc.)

Integrating EEG with other biosignals such as EMG, ECG, and fNIRS has emerged as a promising strategy to enhance the robustness and accuracy of healthcare applications. Multimodal approaches leverage the complementary information contained in different physiological signals, overcoming the limitations of relying solely on EEG, which is often affected by noise, low spatial resolution, and variability. For example, combining EEG and EMG data can improve motor intention detection in neurorehabilitation by capturing both cortical activity and muscle activation, leading to more precise control of assistive devices such as prosthetics and exoskeletons (Pritchard, 2024).

DL architectures, including hybrid CNN-RNN models and graph neural networks, have been developed to effectively fuse multimodal data by learning joint spatial–temporal representations (Xiang, 2024). These models not only improve classification accuracy but also enhance system robustness against artifacts and inter-subject variability. For instance, Grimaldi et al. designed a multimodal DL framework combining EEG, EMG, and accelerometer data for accurate prediction of upper limb movement intention in stroke rehabilitation, achieving superior performance compared to unimodal systems.

Challenges in multimodal integration include synchronization of heterogeneous signals with varying sampling rates, increased computational complexity, and the need for comprehensive multimodal datasets for effective training. Advances in wearable sensor technologies and wireless communication protocols are easing data acquisition and synchronization hurdles, enabling real-time multimodal monitoring in naturalistic settings (Mahato et al., 2024). Overall, the integration of EEG with other biosignals, supported by sophisticated DL models, is paving the way for more reliable and versatile BCI and healthcare monitoring systems. This multimodal paradigm holds significant potential to improve diagnostic accuracy, personalize rehabilitation protocols, and enable seamless human-machine interaction.

### 6.6 Regulatory and ethical considerations

The integration of DL in EEG-based healthcare applications raises critical regulatory and ethical challenges that must be addressed to ensure patient safety, privacy, and equitable access. Medical devices incorporating AI algorithms, especially those used for diagnosis or therapy, require rigorous validation and approval by regulatory bodies. However, the “black-box” nature of many DL models makes it difficult to verify their safety and effectiveness. Regulatory frameworks typically require diagnostic tools to be transparent and explainable (Topol, 2019). As a result, there have been calls to establish standardized guidelines for AI-driven medical devices. These include requirements for continuous monitoring, post-market surveillance, and safeguards against data drift (Ruschemeier, 2023).

Ethical considerations are equally important. The collection and processing of EEG data involve sensitive personal health information, which requires strict compliance with data privacy regulations. Patients should be informed about how their data will be used, stored, and shared, highlighting the need for informed consent and data anonymization. Bias in training datasets also poses a risk, as it can produce AI models that perform unevenly across demographic groups and lead to disparities in healthcare outcomes (Mehrabi et al., 2021). Addressing these issues requires the use of diverse and representative datasets, transparent reporting practices, and continuous fairness assessments. Another ethical dimension concerns the autonomy and agency of patients using EEG-based BCIs. Systems that decode neural activity to control devices or provide neurofeedback must safeguard against misuse or unintended influence, ensuring that users retain full control over their mental states and device operations (Nijboer et al., 2013). The prospect of mind-reading technologies also raises broader societal questions about cognitive liberty and mental privacy, which are still nascent areas in regulatory and ethical discourse.

To foster responsible innovation, multidisciplinary collaboration among clinicians, engineers, ethicists, and regulators is essential. Initiatives promoting open standards, explainable AI, and patient-centric design can facilitate the trustworthy adoption of EEG-based DL systems in healthcare. As the field evolves, proactive engagement with ethical frameworks and regulatory compliance will be key to translating technological advances into safe, effective, and equitable clinical solutions (Morley et al., 2020; Wiens and Shenoy, 2018).

## 7 Conclusion

This comprehensive review has examined the significant advancements in DL approaches applied to EEG-based healthcare applications. We have highlighted the critical role of EEG as a non-invasive, real-time monitoring tool capable of providing rich neural information for diagnosing neurological disorders, enabling brain-computer interfaces, monitoring sleep and fatigue, recognizing mental health states, and facilitating neurorehabilitation. DL architectures such as CNNs, RNNs, LSTMs, transformers, and hybrid models have demonstrated remarkable capabilities in learning complex spatial–temporal patterns from EEG signals, often surpassing traditional signal processing and classical machine learning methods in accuracy and robustness.

The integration of multimodal biosignals, advances in transfer learning, and the emergence of interpretable AI methods have further enhanced the practical utility and clinical relevance of EEG-based DL systems. Nonetheless, challenges remain, including the scarcity of large, diverse, and labeled datasets; substantial inter- and intra-subject variability; computational demands limiting real-time edge deployment; and regulatory and ethical considerations that must be carefully navigated. These limitations underscore the need for continued research focused on model generalization, transparency, lightweight architectures, and the establishment of standardized benchmarks and guidelines.

Despite these hurdles, the potential of DL to revolutionize EEG-based healthcare is immense. It promises more accurate, personalized, and adaptive diagnostic and therapeutic solutions, expanding access to neurotechnologies beyond specialized clinical environments. As interdisciplinary collaborations advance, integrating innovations in sensor technology, algorithm design, and ethical frameworks, EEG-driven DL applications are expected to become indispensable tools in modern medicine. Future efforts must emphasize not only technical improvements but also enhancing trust and accessibility to fully realize the transformative impact of this emerging field.
